# Structural insights and biophysical characterization of p90RSK2:ERK2 complex

**DOI:** 10.1042/BCJ20253110

**Published:** 2025-12-17

**Authors:** Evan Kazuo Kobori, Hoang Phuong My Nguyen, Jian Wu, Katherine Chen, Rodeon Malinovski, Susan Taylor

**Affiliations:** 1 Department of Chemistry and Biochemistry, University of California, San Diego, CA, U.S.A; 2 Department of Pharmacology, University of California, San Diego, CA, U.S.A; 3 Department of Biochemistry and Molecular Biophysics, University of California, San Diego, CA, U.S.A

**Keywords:** AlphaFold modeling, cryogenic electron microscopy, ERK, hydrogen–deuterium exchange mass spectrometry, kinase, p90RSK

## Abstract

Kinase domains are often flanked by flexible tails and intrinsically disordered regions (IDRs) that contain conserved motifs. The co-ordinated action and interplay of IDRs and folded kinase domains is necessary for the proper function of kinases and kinase complexes. Characterization of full-length kinases and complexes is often challenging due to the flexible nature of flanking IDRs, yet necessary to fully understand their function and regulation. The p90 ribosomal S6 kinase (RSK) family is a unique kinase family with two distinct, functional kinase domains (NTK and CTK) flanked by flexible tails and a linker. RSK2 forms a stable complex with its activating kinase, ERK2, and here, we use multiple complementary techniques, hydrogen–deuterium exchange mass spectrometry, cryoelectron microscopy (cryo-EM), and AlphaFold (AF) modeling to study the full-length RSK2:ERK2 complex. We find that broadly, ERK2 is more solvent protected than the NTK/CTK. The NTK N-lobe has quite high deuterium uptake, and analysis of published NTK crystal structures suggests that the NTK N-lobe is dynamic and can adopt a wide range of conformations. The cryo-EM reveals that the RSK2:ERK2 complex adopts a compact shape, and this is consistent with the AF model of the complex, which hints at a possible additional interface between the NTK and ERK2. Collectively, our approach demonstrates that employing multiple complementary techniques can provide insight into the structure and biophysical characteristics of this challenging-to-study kinase complex.

## Introduction

Protein kinases are flexible enzymes that are dynamically regulated between active and inactive conformations [1,2]. Kinase domains are bilobal with the N- and C-lobes acting as independent rigid bodies relative to each other in active kinases. The larger C-lobe is more stable, while the smaller N-lobe is more flexible, capable of sampling a variety of inactive conformations [3]. In most cases, kinase activation is achieved following phosphorylation by an upstream activating kinase. These activator:substrate kinase complexes are mediated by domain:domain interactions and small linear motifs (SLiMs) in intrinsically disordered regions (IDRs) that flank kinase cores [4]. IDRs are typically flank kinase domains, and SLiMs are short flexible sequences that mediate medium affinity interactions, allowing multiple binding partners, depending on the cellular context [5,6]. These are usually conserved motifs that are critical for kinase regulation, kinase dimer interactions, and kinase substrate interaction. The function of these motifs can also be altered by post-translational modifications that alter, either positively or negatively, their binding properties. SLiM/IDR-mediated interactions are dynamic and typically short-lived, making the study of activator-substrate kinase complexes challenging. However, analysis of full-length kinase complexes that include flanking flexible tails is needed to better understand kinase function and how one kinase phosphorylates a substrate kinase.

**Figure 1:**
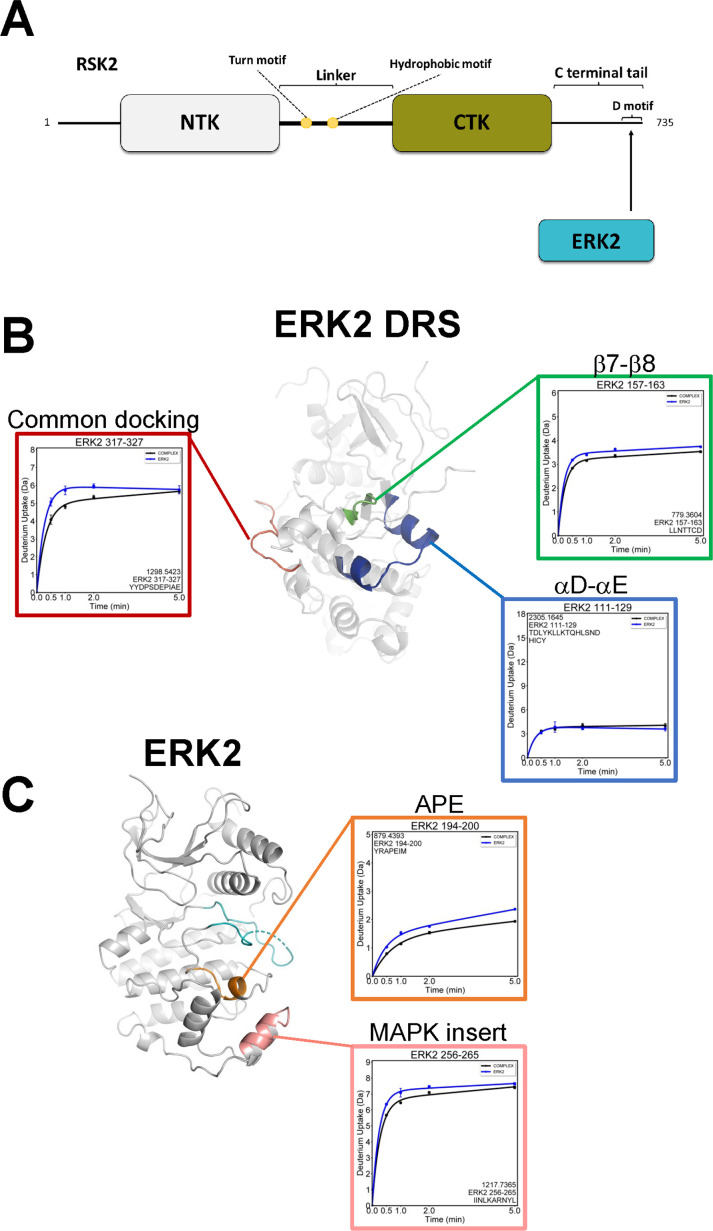
Changes in ERK2 HDX-MS profile upon RSK2 binding. (**A**) Domain organization of the RSK2:ERK2 complex used for HDX-MS studies. (**B**) ERK2 uptake plots at its DRS with and without RSK2. (**C**) Uptake plots of additional ERK2 peptides with changes in uptake upon RSK2 binding. DRS, D-recruitment site; HDX-MS, hydrogen–deuterium exchange mass spectrometry; RSK, ribosomal S6 kinase.

P90 ribosomal S6 kinase (RSK) is a unique family of Ser/Thr kinases; each isoform (RSK1-4) contains two distinct kinase domains and forms a stable complex with its activating kinase ERK1/2 [7,8]. The RSK family comprises four isoforms, each with distinct biological roles due to differential tissue expression and associations with various disease phenotypes. While many studies, including structural analyses, have focused on RSK1, RSK2 has received comparatively less attention. To gain insight into the diversity and biomedical significance of the RSK family, this study focuses on RSK2. RSK contains an N-terminal kinase domain (NTK) and C-terminal kinase domain (CTK), which are members of the AGC and CaMK families, respectively [9,10]. Each kinase domain is flanked by IDRs embedded with SLiMs critical for RSK activation and function [11]. Located at the C-terminal tail of CTK is an autoinhibitory αL helix that suppresses the basal activity of CTK [12], and a reverse orientation of the D (revD) motif that mediates high affinity binding to ERK (Figure 1A) [13,14]. The Linker that connects the NTK and CTK contains the conserved AGC C-terminal extension, which is a hallmark feature of AGC kinases [15] and includes the conserved Ade, Turn, and Hydrophobic motifs (HM). Canonical RSK activation requires four different kinases and its Linker/C-terminal tail. First, ERK2 binds the revD motif and phosphorylates the CTK activation loop, enabling CTK phosphorylation of the HM in the Linker/AGC extension [7,8]. This phosphorylated HM (pHM) then binds PDK1 [16], facilitating phosphorylation of the NTK activation loop and subsequent NTK-mediated phosphorylation of downstream substrates [17–20]. The CTK:ERK2 heterodimer is held together primarily by two distinct interfaces. The flexible CTK C-terminus revD motif (~712–730) binds with high affinity to the D-recruitment site (DRS) of ERK2 (Interface 1). L714 lies in a hydrophobic groove of the DRS, and Args 725,726 form electrostatic interactions with the acidic common docking (CD) site of the DRS (D318, D321, E322). The CTK:ERK2 complex contains a second interface formed by an extended APE-αF helix (on CTK) and the Glycine-rich Loop (on ERK2) (Interface 2). This positions the CTK Activation Loop so that it faces the active site of ERK2, enabling efficient phosphorylation of the Activation Loop. Disruption of Interface 2 via mutation of the CTK APE motif does not cause a significant change in binding affinity, but the CTK becomes a less efficient substrate, while mutation of Interface 1 at either the electrostatic or hydrophobic components prevents DRS site binding [13,21]

There are no structures of FL RSK, and known crystal structures of the isolated NTK and CTK [12,22–27] lack the AGC extension and remaining Linker. The crystal structure of the CTK:ERK2 heterodimer reveals the orientation of the kinase domains of RSK1 and the kinase ERK2 relative to one another and suggests a mechanism for ERK2 phosphorylation of the CTK activation loop in the absence of the NTK and Linker [21]. Thus, structural and biophysical information on a FL RSK2:ERK2 complex is needed to understand the organization of all three kinase domains and their interplay with the flexible tails and linkers for normal RSK function.

Here, we use hydrogen–deuterium exchange mass spectrometry (HDX-MS), cryoelectron microscopy (cryo-EM), and AlphaFold (AF) modeling to obtain biophysical and structural information on the overall size and shape of the full-length RSK2:ERK2 complex. We find that the IDRs of RSK2 that flank the kinase domains: N-terminal, Linker, C-terminal tail have the greatest solvent accessibility in the complex. Among the kinase domains, the N-lobe of the NTK displays the greatest deuterium uptake. Analysis of solved NTK crystal structures reveals that the N-lobe of the inactive NTK is malleable as it can adopt multiple distinct conformations. Both cryo-EM analysis and AF modeling suggest the RSK2:ERK2 complex adopts an overall compact triangular shape, and there may exist an additional interaction between the NTK and ERK2.

## Results

**Figure 2:**
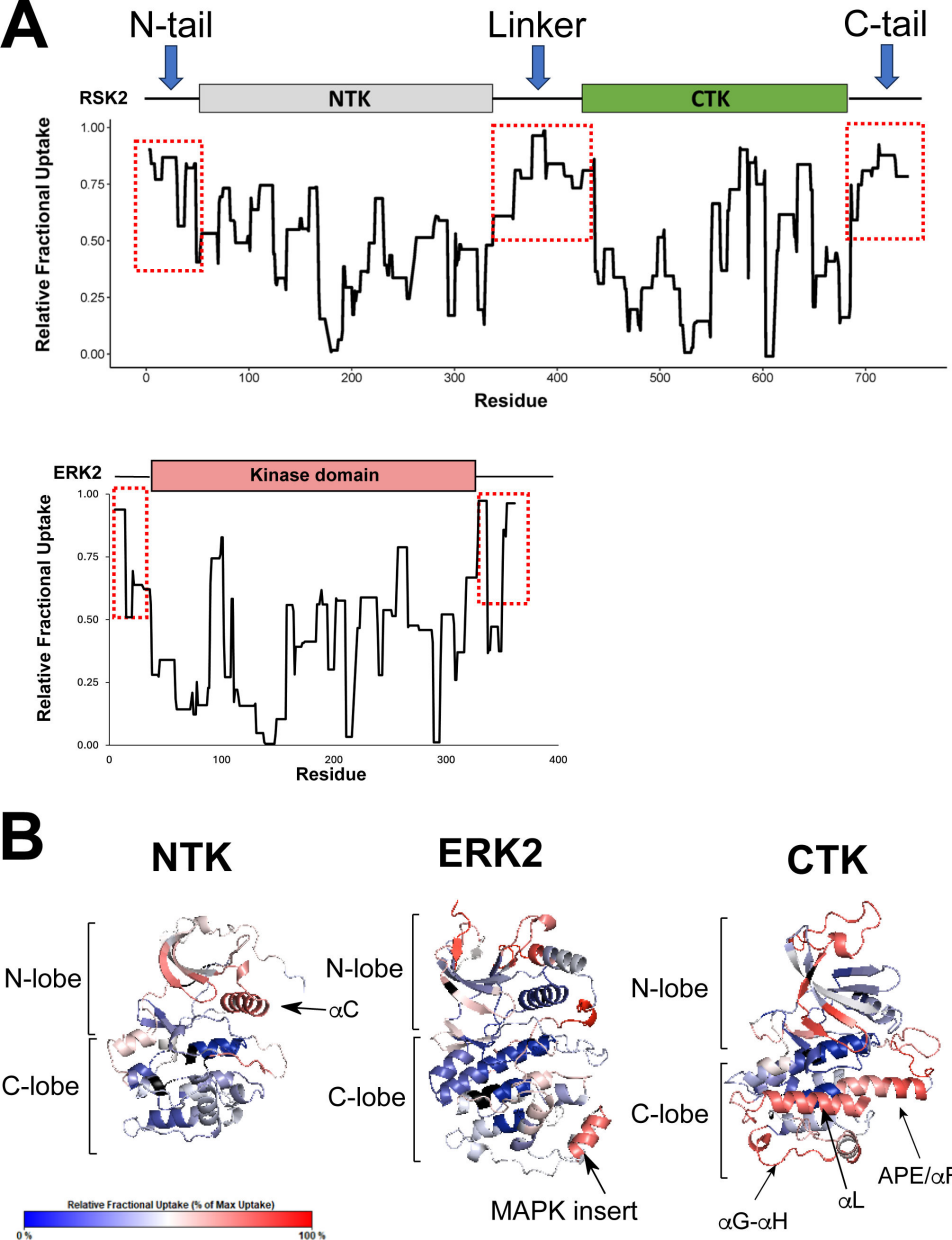
HDX-MS profile of RSK2 in the RSK2:ERK2 complex. (**A**) Per residue fractional uptake of FL RSK2. Uptake for the tails and flexible linker is indicated with red-dashed rectangle. (**B**) Per residue fractional uptake is mapped onto the corresponding kinase domains of RSK2 and ERK2. Relative fractional uptakes are colored as a rainbow scale. HDX-MS, hydrogen–deuterium exchange mass spectrometry; RSK, ribosomal S6 kinase.

In this study, we examined the nonphosphorylated, apo form of the p90RSK2:ERK2 complex in the absence of ATP or ADP, representing a precatalytic initial capture state. To identify flexible regions of the FL RSK2:ERK2 signaling complex, we utilized HDX-MS. We achieved excellent sequence coverage for the entire complex, 99% and 98% for RSK2 and ERK2, respectively, with multiple overlapping peptides (Supplementary Figure S1). First, we compared HDX profiles of free ERK2 and ERK2 with RSK2 bound and observed modest protection at the conserved ERK2 DRS site, both at the electrostatic common docking site, and the β7- β8 loop that forms the hydrophobic groove (Figure 1B, Supplementary Figure S2A). We also observed additional modest, yet statistically significant, protection at the ERK2 DFG phenylalanine, APE motif, and MAPK specific insert (Figure 1C, Supplementary Figure S2B). This protection may be due to a direct interaction with RSK2 or indirect conformational changes. ERK2 binding to D motifs is known to stabilize the active site, and the MAPK insert mediates protein:protein or protein:nucleic acid interactions [28]. For the RSK2:ERK2 complex, the unstructured regions flanking the kinase domains have the greatest solvent exposure, particularly the N-tail, Linker, and C-tail of RSK2 (Figure 2A). These are predicted to be intrinsically disordered (Supplementary Figure S1) and are the most dynamic and flexible regions in the complex. Within each kinase domain, the core hydrophobic helices such as the αE, αF, and αH helices are well shielded from solvent (Supplementary Figure S3A). Regions that contribute to nucleotide binding such as the hinge, Gly-rich loop, and activation segments are all well solvent exposed, as expected in a nonphosphorylated, apo sample (Supplementary Figure S3B,C,D).

**Figure 3:**
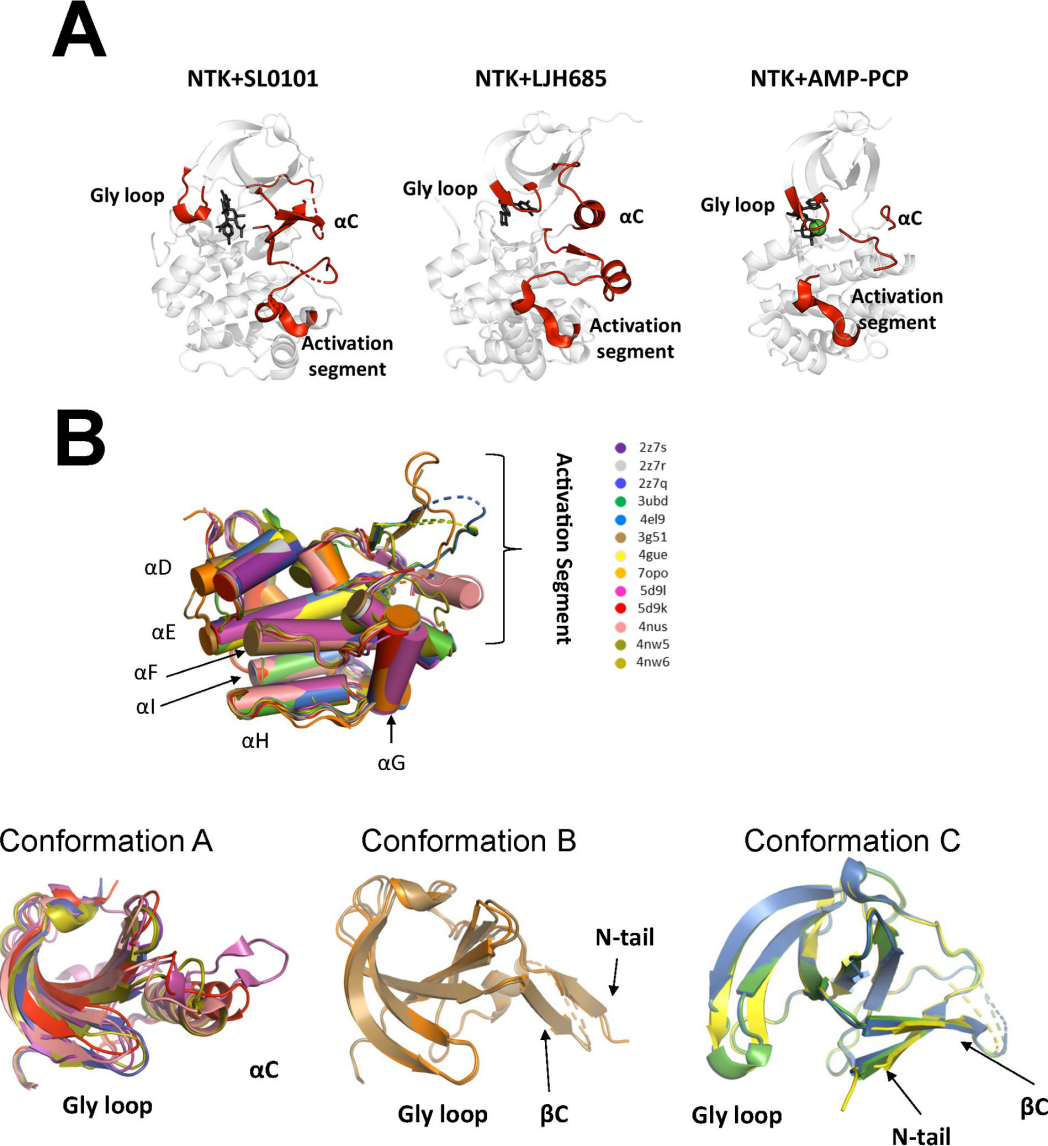
NTK N-lobe conformational diversity. (**A**) Crystal structures of the NTK bound to various inhibitors with the corresponding Gly loop, αC, and activation segments colored red. From left to right, PDB IDs: 3ubd, 4nus, 2z7q. (**B**) Aligned C-lobes and activation segments of NTK structures, top. Bottom, NTK N-lobes are sorted into three distinct Conformations: A, B, and C. NTK, N-terminal kinase.

By comparing the HDX profiles of all two kinase domains of RSK2 and kinase ERK2, we found that ERK2 is more protected from solvent than the other two kinase domains. The N-lobe of the NTK is considerably more solvent exposed than the corresponding N-lobes of CTK or ERK2 (Figure 2B). In addition to the Gly loop and ATP binding hinge region, the b3-aC loop, αC, b5, and aD helix are all solvent exposed (Supplementary Figure S3B), suggesting that the N-lobe is highly dynamic and conformationally malleable. To explore the conformational dynamics of the NTK, we compared all of the publicly available crystal structures of the NTK bound to various nucleotides and inhibitors [22–27] (Figure 3A). The C-lobes are well aligned, regardless of isoform or inhibitor, while the activation segments and N-lobes are poorly aligned and dynamic (Figure 3B). These structures all lack the AGC extension, and we identified that the NTK N-lobes adopt one of three major conformational states, Conformations A, B1, and B2, which can be distinguished by the secondary structure and position of their αC helices (Figure 3B). Conformation A structures feature a helical, intact αC helix in more of a canonical, ‘in’ position. RSK1 NTK structures are considered as Conformation A despite an unfolded or disordered αC [24], since no other residues occupy this position. Other structures, which we call Conformations B and C, contain rather extreme secondary structure perturbations to the αC helix that result in the formation of an unusual β sheet (hereafter referred to as βC) that replaces the αC helix [26,27]. The β9 strand of the activation segment, along with the βC, replaces the position of the canonical αC helix in B1 structures, while in B2 structures, the βC, in concert with an N-terminal β strand, replaces the αC helix. The C conformation is also characterized by a distortion of the glycine-rich loop, where it is rotated out away from the active site cleft.

These NTK structures (PDB: 4nw5, 3g51, 3ubd, 4nus) also adopt unique internal hydrophobic spine architectures (Supplementary Figure S4). The C spines of all NTK structures are similar across all conformations, while the R spines are more dynamic [29,30]. In the B2 conformation, the R spine is broken, indicating that this is an inactive conformation, completely unable to phosphorylate heterologous substrates. The B1 R spine is more of an intermediate case. While the spine is contiguous, it experiences a branching at RS3 (L122) and RS4 (L133) that is not typical for canonical active AGC kinases, suggesting that this may be some type of an intermediate state. The R spines are variable in Conformation A, broken when the RSK2 NTK is bound to the inhibitor LJH685 [22] (PDB: 4nus) but intact when bound to a benzoxazole compound [23] (PDB: 4nw5, 4nw6). Thus, some Conformation A structures better resemble an active-like AGC kinase conformation although in all cases the AGC extension is missing and the C-spine is incomplete. This analysis of NTK structures and conformations in combination with the HDX-MS demonstrates the flexibility and malleability of the NTK. The flexible nature of the NTK allows various inhibitors to stabilize the NTK in inactive (B2), intermediate (B1), and active-like (A) conformations and highlight its extreme malleability.

The C-lobe of the CTK is more solvent exposed than its N-lobe or the corresponding C-lobes of the NTK or ERK2. Specifically, the αG-αH, long extended APE/αF, and autoinhibitory αL helices exhibit greater solvent exposure compared with the rest of the kinase domains (Figure 2B). The αG and αG-αH loops have higher B-factors compared with other parts of the kinase domain in the CTK:ERK2 heterodimer crystal structure, suggesting that this part of the CTK has some flexibility (Supplementary Figure S5A) consistent with the αG helix role in substrate recognition [31]. Intriguingly, the B-factors of the extended APE/αF helix are quite low, despite the strong deuterium uptake and this region being predicted to be partially unfolded (Supplementary Figure S5A,B). Thus, in solution, this extended APE/αF may be more of a weak helix than is observed in the CTK-ERK2 crystal structure. The CTK potentially has some basal activity and can autophosphorylate the hydrophobic motif in the absence of ERK2 phosphorylation [32]. Moreover, we observed basal pHM in overexpressed WT RSK2, RSK2 K100R (NTK kinase dead mutant), but not in RSK2 K451A (CTK kinase dead mutant) (Supplementary Figure S5C). Therefore, the strong uptake of the autoinhibitory αL helix may be due to dynamic binding of this helix to the CTK active site (Supplementary Figure S5A,B) as hypothesized in [4].

**Figure 4:**
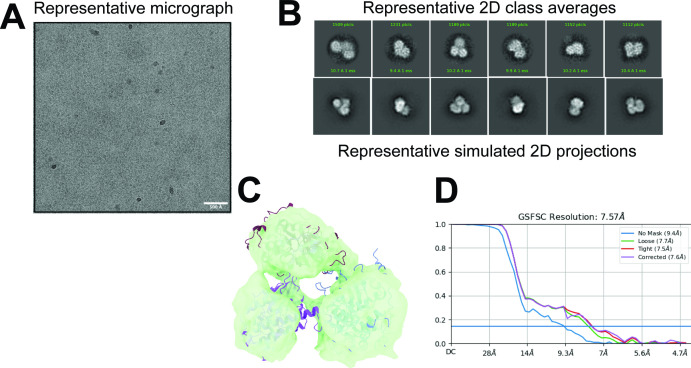
Structural analysis of the RSK2:ERK2:ORF45 complex. (**A**) Representative micrograph of RSK2:ERK2:ORF45 complex (**B**) Comparison of experimental 2D class average and projections from AF-predicted models (**C**) 3D reconstruction of RSK2:ERK2:ORF45 complex (**D**) FSC curve of the refined map. AF, AlphaFold; RSK, ribosomal S6 kinase.

We elucidate the overall size, shape, and organization of the RSK2:ERK2 complex by combining cryo-EM and AF predictions. These complementary approaches enabled us to capture structural features and gain insights into the dynamics of the complex. To enhance the stability of the RSK2:ERK2 complex during sample preparation, we incorporated the viral peptide ORF45, which has been previously demonstrated to bind both RSK2 NTK and ERK2 simultaneously [33], thereby facilitating complex stabilization. The cryo-EM data revealed well-distributed particles across the micrographs (Figure 4A), and subsequent 2D classification generated well-defined secondary structural features that closely mirrored the projections of our simulated AF models (Figure 4B). After extracting 316,071 particles from 1025 micrographs, we performed multiple rounds of 2D classification, selecting those classes that most closely resembled the simulated projections. This iterative refinement process ensured the selection of particles that contained distinct features reflective of the trimeric RSK2:ERK2 complex. The *ab initio* reconstruction and homogeneous refinement process culminated in a 7.8 Å cryo-EM map (Figure 4C and D, Supplementary Figure S6A, and S7), which revealed a compact, triangular shape for the RSK2:ERK2 complex. Notably, this reconstruction provides the first clear structural insight into the spatial arrangement of the kinase domains of RSK2 and ERK2, with distinct densities corresponding to the individual kinase domains (Figure 4C, Supplementary Figure S6A).

**Figure 5:**
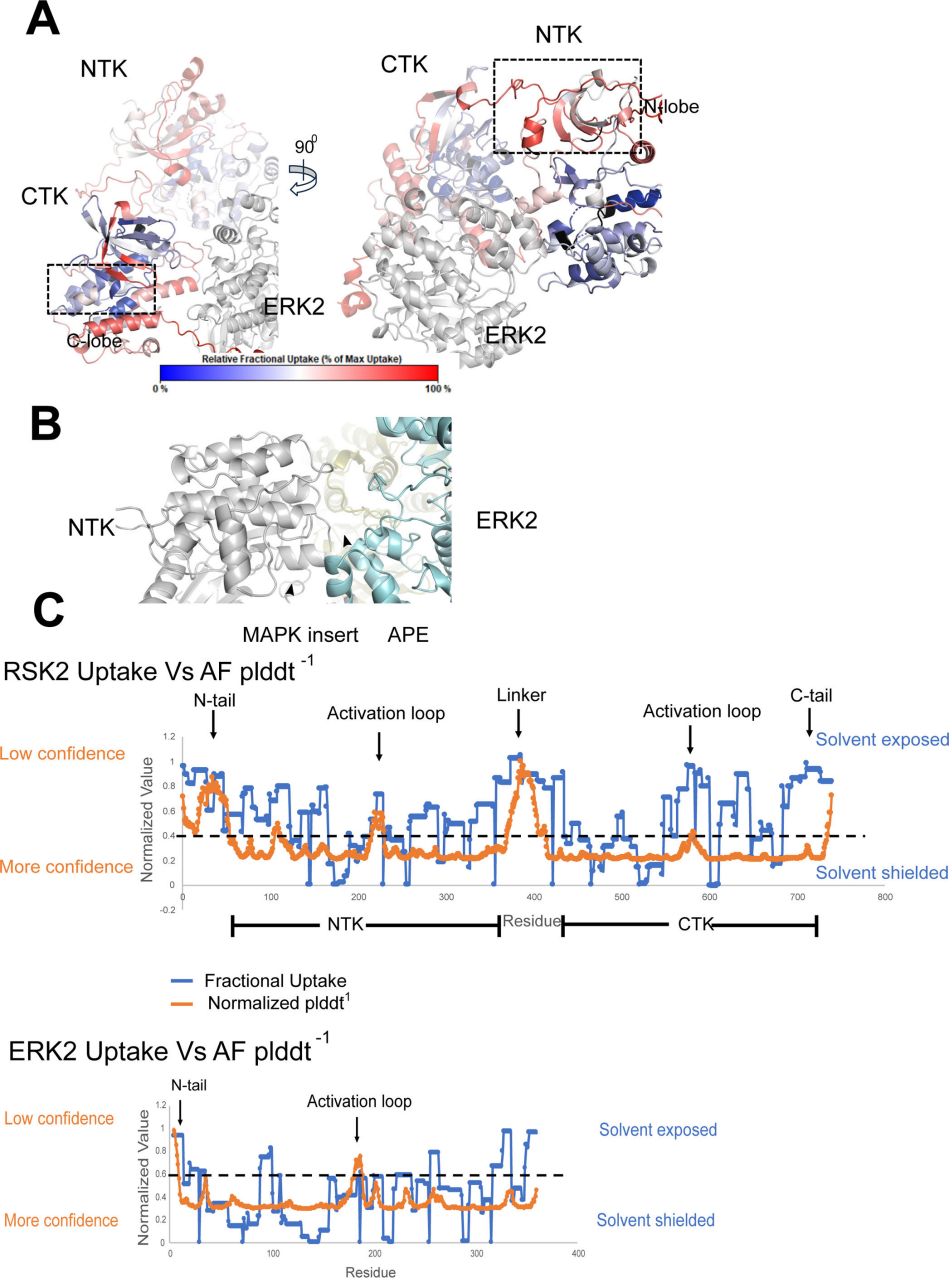
AlphaFold modeling of the RSK2:ERK2 complex. (**A**) AlphaFold model of RSK2:ERK2 complex with RSK2 colored as HDX-MS deuterium uptake values as b-factors. (**B**) RSK2:ERK2 AlphaFold model showing an additional interface between the NTK C-lobe and ERK2 MAPK insert and APE motif. NTK in gray, CTK in green, and ERK2 in cyan. (**C**) Comparison of plddt score and uptake value of RSK2 (top) and ERK2 (bottom). CTK, C-terminal kinase; HDX-MS, hydrogen–deuterium exchange mass spectrometry; NTK, N-terminal kinase.

Given the lack of a full-length structure for the RSK2:ERK2 complex, we used AF to predict the complete structural model of the complex. By utilizing sequence data from Uniprot and generating five distinct AF models, we observed structural diversity in the termini, linkers, and loops, although all models shared a consistent core structure and an overall compact trimers. The highest-ranked AF model was chosen for further analysis (see Methods), and its predicted structure generally aligned well with the cryo-EM density map (**Figures 4B, C, 5A**, Supplementary Figure S6). Both the cryo-EM and AF-derived structures adopt a compact, triangular configuration, underscoring the robustness of our findings across complementary methods. The AF model provided valuable insights into the flexibility of the complex, particularly in regions such as the intrinsically disordered N-term, activation loop, linker, and C-tail of RSK2, and the N-term and activation loop of ERK2 (Figure 5C). The AF model did not model conformation B and B1 on NTK, though the confidence scores at the aC are low (Figure 5A and C, Supplementary Figure S6B). These flexible regions correspond to areas of low confidence in the AF model, consistent with the findings from HDX-MS, which showed that these regions are more solvent-exposed and undergo more conformational flexibility.

Our combined structural analysis allowed us to reconcile the cryo-EM and AF models with HDX-MS data, which revealed important details about the solvent accessibility and structural dynamics of the RSK2:ERK2 complex. Specifically, the HDX data indicated that the C-lobe of the CTK and the N-lobe of the NTK are both solvent-accessible and unimpeded by the other kinase domains, suggesting that these regions may be involved in downstream interactions or regulatory events (Figure 5A and B). This is consistent with the structural interpretation of the cryo-EM map and the AF model.

Interestingly, our model also suggests a potential tertiary interaction between the ERK2 MAPK insert and the APE motif with the C-lobe of the NTK (Figure 5B). This interface, although modest in terms of protection as observed by HDX, provides evidence for an additional but minor interaction between ERK2 and RSK2, further stabilizing the overall complex and possibly contributing to the functional cross-talk between the two kinases.

## Discussion

The p90 RSK kinases with their multistep activation pathway are defined by their two distinct kinase domains, in addition to three IDRs with critical SLiMs [7,8]. The concerted action of the NTK, CTK, ERK2, and PDK1, the RSK Linker and C-terminal tail, co-ordinates the generation of an active NTK. Thus, further characterization of this pathway can provide a greater understanding of how IDRs and kinase domains collectively regulate kinase function [4]. Here, we performed HDX-MS, AF modeling, and cryo-EM on the stable precatalytic FL RSK2:ERK2 kinase complex to explore the dynamic or unstructured regions, and the overall size and shape of the complex.

HDX-MS reveals that the IDR regions flanking all three kinase domains, in particular the N-terminal segment, Linker, and C-terminal tail of RSK2 have the highest solvent exposure and are the most dynamic regions in the complex. The N-terminal of RSK is poorly conserved among the four RSK isoforms and has unknown function. Similar N-terminal tails of other AGC kinases can aid in kinase thermal stability and cellular targeting [34]. The N-terminus of RSK3 contains a putative nuclear localization signal [35], and RSK2 N-terminal deletion constructs are more susceptible to precipitation [26]. Thus, the N-terminal tail in RSK may have similar functions in localization and/or thermal stability. The strong solvent exposure and flexibility of the Linker and AGC extension, particularly for the HM, is necessary to facilitate the various steps in RSK activation. The HM serves as a good example of a versatile SLiM capable of different interactions depending on the biochemical and cellular environment [4,36,37]. The HM is a substrate for the CTK, a docking site and a *trans* activator of PDK1, and the HM stabilizes the NTK active conformation by filling the NTK PIF pocket, anchoring the AGC extension to the N-lobe [7,8,15,38]. The RSK2 CTK C-terminal tail is strongly solvent exposed as well, but this may be due to the lack of secondary structure and flexibility around the ERK2 binding revD motif, rather than instability of this interface. The C-terminal tail has no visible density in structures of the isolated CTK; ERK2 binding orders the C-terminal tail and drives the high-affinity binding [12,21].

All three kinase domains displayed solvent protection/exposure patterns similar to other analyzed kinase domains [39]. The modest solvent exposure observed in the C-lobe, particularly the aG-aH, aH-aI loops for all three kinase domains is indicative of their role in mediating protein:protein interactions. PKA, for example, utilizes this surface for additional interactions with its various Regulatory (RI/RII) subunits [40] as well as for its interactions with the heat stable protein kinase inhibitor [40–42]. The aH-aI loop can also serve as a distal tethering site for other substrates [43]. Similarly, the MAPK insert of ERK2 mediates nucleic acid and other protein binding interactions [28]. We propose that this site is part of a minor interface with the NTK C-lobe as predicted by our AF modeling and modest observed HDX protection upon RSK2 binding. Generally, the interaction between the RSK C-terminal tail and the D-recruitment site (DRS) of ERK2 drives high affinity binding; thus, this potential interface involving the MAPK insert likely has another role. Nonetheless, this minor additional interface and the overall compact shape of the complex demonstrate that despite the flexible linker separating the NTK and CTK, the NTK is part of a compact complex and is likely not acting independently of both ERK2 and the CTK.

ERK2 and the CTK are better shielded from solvent than the NTK, suggesting that these kinase and kinase domain sample fewer conformational states. The N-lobe of the NTK is more malleable, capable of sampling a wide conformational space. Recently, an analysis of the conformational space of the LRRK2 kinase domain indicates that different inhibitors can stabilize the kinase domain into distinct conformational states [44]. We made similar observations here. The NTK adopts two major conformational states that are differentiated by the secondary structure and position of the αC helix, depending on the type of bound inhibitor. The αC helices are known to be mobile, capable of moving between ‘in’ and ‘out’ conformations depending on the activation state of the kinase [1,2,45]. However, we are not aware of any other kinase that refolds its αC into a βC strand as in NTK Conformation B structures. All analyzed NTK structures lack the AGC extension or an ordered phosphorylated activation loop, and most contain broken R spines. A few Conformation A (pdb:4nw5, 4nw6) structures are in ‘active-like states’ with intact R spines and mostly properly positioned catalytic and regulatory residues. These active-like structures suggest that assembly of an R spine does not necessarily require activation loop phosphorylation nor an AGC extension. Moreover, activation loop phosphorylation and AGC extension anchoring appear to be the final steps for the assembly of an active kinase.

The HDX profile of the CTK is a bit unusual as most kinase domains feature a stable C-lobe and a more solvent-exposed, flaky N-lobe, like the NTK. The kinase domain of LRRK2 has a similar HDX profile, where its N-lobe is stabilized by interactions with other neighboring domains, leaving its C-lobe more solvent exposed [44]. The CTK is an intriguing kinase domain due to its highly specialized nature and unusual long, extended APE/αF helix that is observed in both the presence and absence of ERK2. The HM is the only known substrate of the CTK, thus following CTK activation loop phosphorylation, only displacement of αL and rearrangement of the extended APE/αF helix are needed to allow access to the active site and arrangement of a *P*+1 substrate docking loop [12].

Despite promising, featureful 2D class averages, our initial cryo-EM analysis of the RSK2:ERK2 complex was limited to low resolution. In addition to having a relatively small dataset and particle stack, this particular complex is challenging due to the similarity of three kinase domains, which we suspect creates a pseudosymmetry difficult for particle alignment. Additionally, the FSC curve suggests that having a larger dataset with more particles may help to obtain higher resolution (Figure 4D). Thus, higher resolution reconstructions of this complex may be possible with a significantly larger dataset, or with an additional binding partner to break the inherent pseudosymmetry.

Our structural and computational analyses of the RSK2:ERK2 complex have provided a comprehensive view of its organization and functional dynamics. Through cryo-EM and AF modeling, we have demonstrated that the RSK2:ERK2 complex adopts a compact, triangular conformation, with clear structural features that correspond to the individual kinase domains of both proteins. This work provides the first direct structural evidence of the kinase domain organization in the RSK2:ERK2 complex and offers important insights into how these two kinases might function together. The use of AF predictions in combination with cryo-EM data has been pivotal in understanding regions of flexibility and disorder within the complex, specifically in the terminal regions of both RSK2 and ERK2. These findings highlight the dynamic nature of the RSK2:ERK2 complex, particularly in regions that are exposed to the solvent and may be involved in regulatory or protein-protein interactions. Our analysis also suggests a minor yet potentially significant tertiary interaction between ERK2 and RSK2, which could play a role in modulating the functional cross-talk between the kinases. The identification of this interaction provides new directions for future studies, potentially leading to a better understanding of the regulatory mechanisms governing RSK2 and ERK2 signaling pathways.

In conclusion, this study not only advances our understanding of the structural features of the RSK2:ERK2 complex but also lays the groundwork for future investigations into its biological relevance and therapeutic potential. Our findings open the door to exploring how small-molecule inhibitors or modulators of the RSK2:ERK2 interaction might be designed to disrupt or modulate the signaling pathways in which these kinases are involved, with implications for cancer, neurobiology, and other diseases.

## Methods

### Protein expression and purification

Proteins were expressed in either *E. coli* BL21 Rosetta pLysS in pET vectors. Human full-length (FL) RSK2, EKR2, and ORF45 were expressed and purified as described previously. Briefly, FL RSK2 was expressed with a Tev cleavable N-terminal MBP tag and a non-cleavable C-terminal His tag. A Tev cleavable His-tagged ERK2 was coexpressed with lambda phosphatase for generating inactive ERK2. ORF45 (16-76) was expressed with an N-terminal GST tag [29]. Following transformations, all proteins were grown in LB at 37° until OD:0.6, then the temperature was reduced to 18° and IPTG was added to 0.1 mM. Expression continued overnight, and pellets were collected and frozen.

Cell pellets were resolubilized in lysis buffer (50 mM Tris, pH~8.0, 200 mM NaCl, 5 mM βME, 5% glycerol, 1 mM Benzamidine, 1 mM AEBSF), lysed by a microfluidizer, and clarified lysate was added to equilibrated Ni resin. Ni resin for RSK2 and ERK2 was washed with lysis buffer supplemented with 10 mM imidazole and eluted with lysis buffer supplemented with 200 mM imidazole. Ni eluted RSK2 was then added to equilibrated amylose resin, washed with lysis buffer, and eluted with 20 mM Tris pH~8.0, 200 mM NaCl, 2 mM DTT, 5% glycerol, 10 mM maltose. Eluted ERK2 and RSK2 were then dialyzed overnight in low salt buffer (20 mM Tris pH~8.0, 50 mM NaCl, 2 mM DTT, 5% glycerol) with Tev protease. ERK2 and RSK2 were further purified by anion exchange using a Hitrap Q column to isolate non-phosphorylated ERK2 or free RSK2, followed by SEC with a Superdex 200 16/60.

Cell pellet for ORF45 was resolubilized in lysis buffer, lysed by a microfluidizer, and clarified lysate was applied to equilibrated glutathione resin. The resin was washed with lysis buffer and eluted with lysis buffer supplemented with 10 mM free glutathione. Eluted ORF45 was dialyzed overnight in low salt buffer with Tev protease and then further purified by SEC using a Superdex 200 16/60.

RSK2:ERK2 or RSK2:ERK2:ORF45 complexes were prepared by mixing purified RSK2, ERK2, or ORF45 proteins in a 1:1.3, or 1:1.3:1.5 molar ratio, respectively, and then purified by SEC. (Supplementary Figure S8).

### Hydrogen–deuterium exchange mass spectrometry

HDX-MS was performed as described previously, at the Biomolecular and Proteomics Mass Spectrometry Facility (BPMSF) of the University of California San Diego, using a Waters Synapt G2Si system with HDX technology (Waters Corporation) according to methods previously described. The binary RSK2:ERK2 complex was subjected to HDX-MS analysis, and results of this were compared with that of ERK2 or RSK2:ERK2:ORF45 (16-76) sample. Briefly, deuterium exchange reactions were performed using a Leap HDX PAL autosampler (Leap Technologies, Carrboro, NC). D_2_O buffer was prepared by lyophilizing gel filtration buffer, (20 mM Hepes pH 7.5, 150 mM NaCl, and 1 mM TCEP) initially dissolved in ultrapure water and redissolving the powder in the same volume of 99.96% D_2_O (Cambridge Isotope Laboratories, Inc., Andover, MA) immediately before use. Deuterium exchange was measured in triplicate at each time point (0 min, 0.5 min, 1 min, 2 min, 5 min). For each deuteration time point, 4 μl of protein was held at 25°C for 5 min before being mixed with 56 μl of D_2_O buffer. The deuterium exchange was quenched for 1 min at 1°C by combining 50 μl of the deuteration reaction with 50 μl of 3M guanidine hydrochloride, final pH 2.66. The quenched sample (90 μl) was then injected in a 100 μl sample loop, followed by digestion on an in-line pepsin column (Immobilized Pepsin, Pierce) at 15°C. The resulting peptides were captured on a BEH C18 Vanguard precolumn, separated by analytical chromatography (Acquity UPLC BEH C18, 1.7 µm 1.0 × 50 mm, Waters Corporation) using a 7–85% acetonitrile gradient in 0.1% formic acid over 7.5 min, and electrosprayed into the Waters Synapt G2Si quadrupole time-of-flight mass spectrometer. The mass spectrometer was set to collect data in the Mobility, ESI + mode; mass acquisition range of 200–2000 (m/z); scan time 0.4 s. Continuous lock mass correction was accomplished with infusion of leu-enkephalin (m/z = 556.277) every 30 s (mass accuracy of 1 ppm for calibration standard).

For peptide identification, the mass spectrometer was set to collect data in mobility-enhanced data-independent acquisition (MS^E^), mobility ESI + mode instead. Peptide masses were identified from triplicate analyses, and data were analyzed using the ProteinLynx global server (PLGS) version 3.0 (Waters Corporation). Peptide masses were identified using a minimum number of 250 ion counts for low energy peptides and 50 ion counts for their fragment ions; the peptides also had to be larger than 1,500 Da. The following cutoffs were used to filter peptide sequence matches: minimum products per amino acid of 0.2, minimum score of 7, maximum MH+error of 5 ppm, and a retention time RSD of 5%. In addition, the peptides had to be present in two of the three ID runs collected. The peptides identified in PLGS were then analyzed using DynamX 3.0 data analysis software (Waters Corporation). Peptides containing mutated residues were manually assigned. The relative deuterium uptake for each peptide was calculated by comparing the centroids of the mass envelopes of the deuterated samples with the undeuterated controls following previously published methods. The peptides reported on the coverage maps are actually those from which data were obtained. For all HDX-MS data, three technical replicates were analyzed. Data are represented as mean values ± SEM of three technical replicates due to processing software limitations; however, the LEAP robot provides highly reproducible data for biological replicates. The deuterium uptake was corrected for back-exchange using a global back-exchange correction factor (typically ~25%) determined from the average percent exchange measured in disordered termini of various proteins. ANOVA analyses and *t* tests with a *P* value cutoff of 0.05 implemented in the program, DECA, were used to determine the significance of differences between HDX data points. Deuterium uptake plots were generated in DECA (github.com/komiveslab/DECA), and the data are fitted with an exponential curve for ease of viewing.

### Cross-linking

RSK2:ERK2:ORF45 complexes were further stabilized by chemical cross-linking by incubation with glutaraldehyde. Glutaraldehyde was diluted in cross-linking buffer (50 mM Hepes pH 7.5, 150 mM NaCl, 1 mM TCEP) to a 5% stock solution. Purified RSK2:ERK2:ORF45 complex was diluted in cross-linking buffer to 0.1 mg/ml and was cross-linked by addition of diluted glutaraldehyde 0.1 % v/v. The reaction proceeded for 10 min at room temperature, and cross-linking was quenched by addition of 100 mM Tris pH 8, and the sample was placed on ice. The sample was then concentrated and applied to an S200 gel filtration column to separate cross-linked RSK2:ERK2:ORF45 complex from overcross-linked higher molecular weight artifacts (Supplementary Figure S8).

### Cryo-EM sample preparation, data collection, and data analysis

Purified samples that were stabilized by cross-linker or without were applied to plasma cleaned UltrAufoil 1.2/1.3 gold grids. Grids were prepared using a Vitrobot and stored in liquid nitrogen until imaged. Data acquisition for the RSK2:ERK2:ORF45 was carried out at UCSD’s cryo-EM Facility on a Titan Krios G3 (Thermo Fisher Scientific) operating at 300 keV equipped with a Gatan BioContinuum energy filter. Images were collected at a magnification of 130,000× in EF-TEM mode (1.09 Å calibrated pixel size) on a Gatan K2 detector using a 20 eV slit width and a cumulative electron exposure of ~44 electrons/Å^2^ (50 frames). Data were collected automatically using EPU with aberration-free image shift using a defocus range of −0.5 to −4 µm. Using Cryosparc, raw movies were motion and CTF corrected using Patch Motion and Patch CTF correction jobs. Micrographs with CTF fits worse than 4.5 Å were discarded. Picked particles were extracted. A total of 316,071 particles were extracted from 1025 micrographs, downsampled 2 × 2 (2.18 Å/pixel, 128 pixel box size) and imported into cryoSPARC and subjected to 2D classification. Those 2D class averages containing the strongest secondary structural details and representing intact RSK2, ERK2, and ORF45 were selected (40,611 particles in total). These particles are subjected to *ab initio* reconstruction with C1 symmetry and homogeneous refinement. A 7.57 Å structure was obtained.

### AF modeling

Models of the FL RSK2:ERK2 complex were generated using the ColabFold multimer server. Sequences for RSK2 and ERK2 were Uniprot accession numbers P51812 and P28482, respectively. The highest ranked model was selected for further analysis.

## Supplementary material

online supplementary material 1.

## Data Availability

The authors confirm that the data supporting the findings of this study are accessible within the article and its supplementary materials. Any additional requests can be directed to the corresponding author.

## Open Access

Open access for this article was made possible by the University of California’s participation in an inclusive Read & Publish agreement with Portland Press and the Biochemical Society, as part of a transformative deal with UC.

## Abbreviations

AGC: kinase family including PKA, PKG, and PKC;
APE: Alanine-Proline-Glutamate motif
CTK: C-terminal kinase
CaMK families: calcium/calmodulin-dependent protein kinase
DFG: Aspatate–Phenylalanine–Glycine motif
DRS: D-recruitment site
ERK2: extracellular signal-regulated kinase
HDXMS: hydrogen–deuterium exchange mass spectrometry
HM: hydrophobic motifs
IDRs: intrinsically disordered regions
LRRK2: leucine-rich repeat protein kinase 2
MAPK: mitogen-activated protein kinase
NTK: N-terminal kinase
ORF45: open reading frame 45 protein
PDK1: 3-phosphoinositide-dependent protein kinase 1
PKA: protein kinase A
RI/RII: regulatory subunits I/II
SLiMs: small linear motifs
cryoEM: cryogenic electron microscopy
pHM: phosphorylated HM
p90RSK: p90 ribosomal S6 kinase
revD: reverse orientation of the D motif
