# Supplementary material for: Structural insights and biophysical characterization of p90RSK2:ERK2 complex
Source: Biochem J. 2025 Dec 17;482(24):1831–43. doi: 10.1042/BCJ20253110 (PMC12614931; doi:10.1042/BCJ20253110)
Supplement: online supplementary material 1. [file bcj-482-24-BCJ20253110-s001.docx]

**Supplemental figures**

| 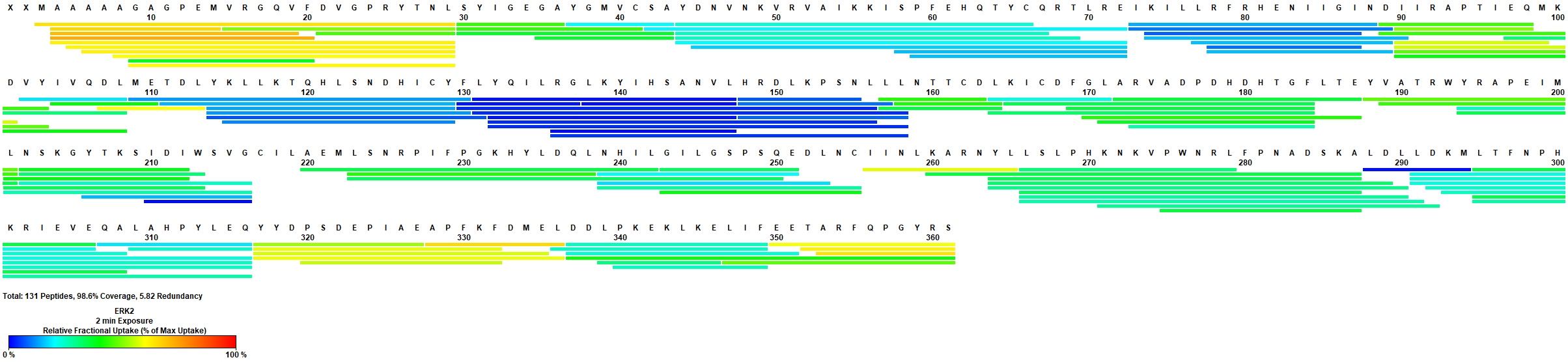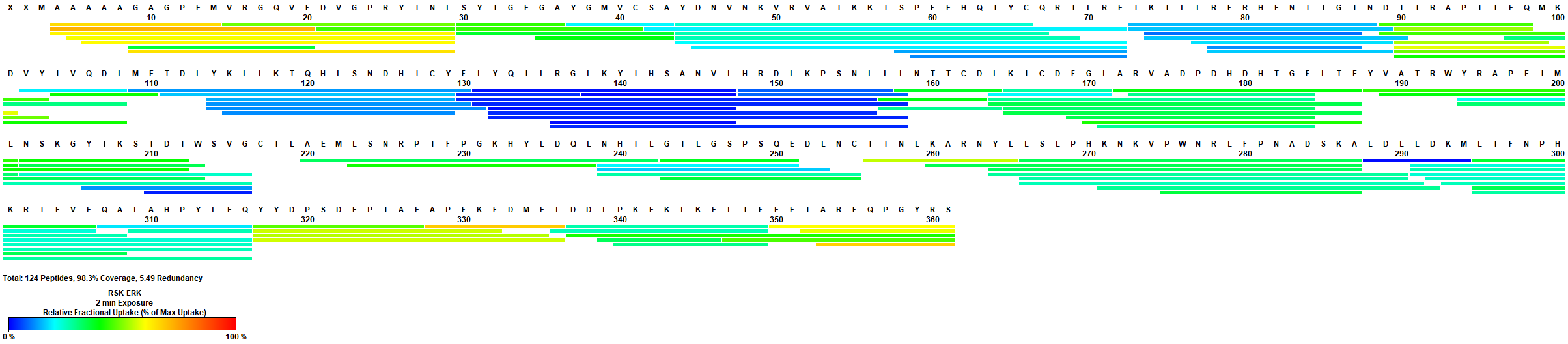 **Common Docking**  **C-terminus**  **N-terminus**  **β1**  **β2**  **β3**  **β4**  **αC**  **β5**  **αD**  **αE**  **β6**  **β7**  **β8**  **β9**  **αF**  **αG**  **αH**  **αI**  RSK2**:ERK2**  **Common Docking**  **C-terminus**  **N-terminus**  **β1**  **β2**  **β3**  **β4**  **αC**  **β5**  **αD**  **αE**  **β6**  **β7**  **β8**  **β9**  **αF**  **αG**  **αH**  **αI** 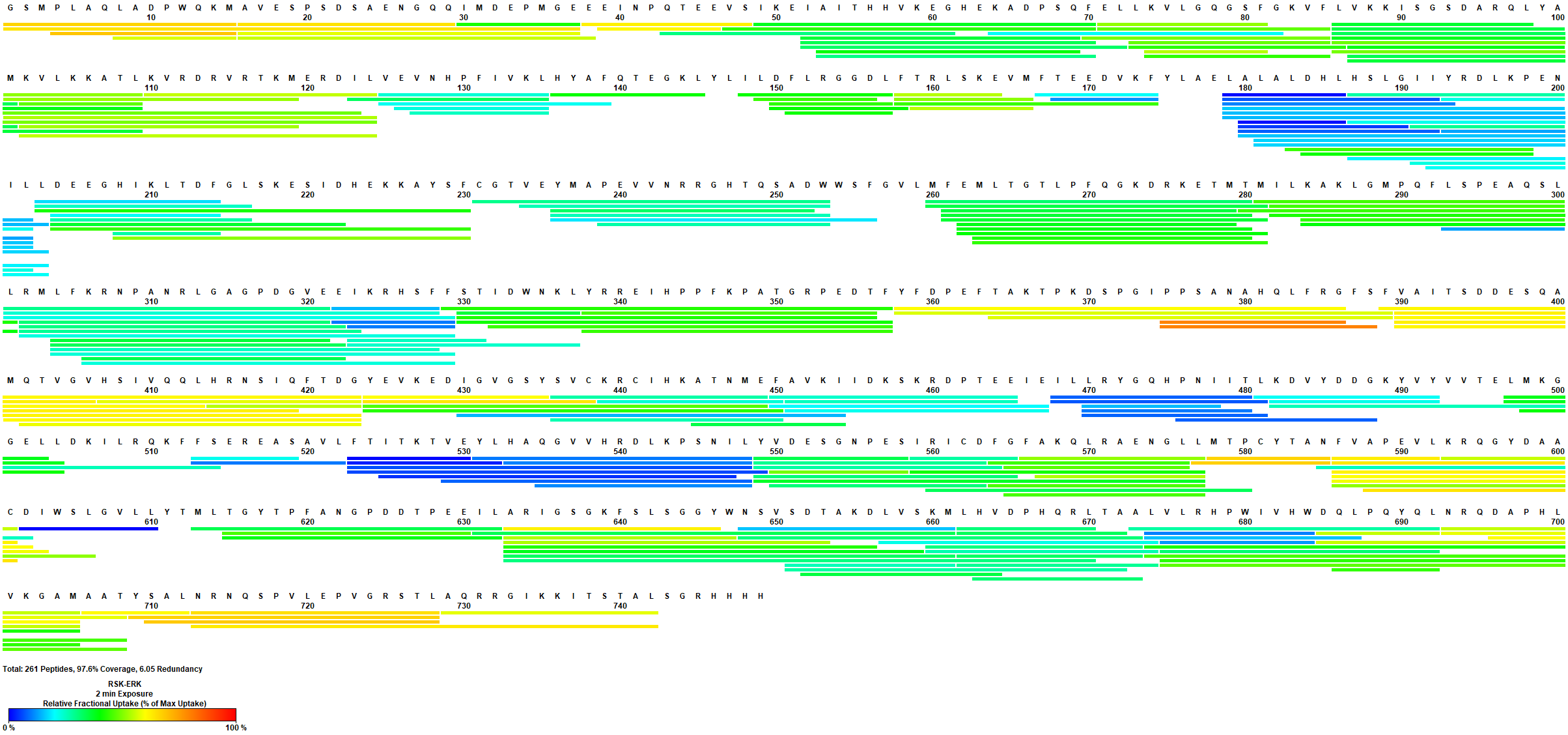 **NTK**  **N-terminal Tail**  **AGC Tail/Linker**  **Linker**  **CTK**  **C-terminal Tail**  **β1**  **β2**  **β3**  **β4**  **β3**  **αC**  **αD**  **β5**  **αE**  **β6**  **β7**  **β8**  **αF**  **αG**  **αH**  **αH**  **αI**  **β1**  **β2**  **β3**  **αC**  **β4**  **β5**  **αD**  **αE**  **β6**  **β7**  **β8**  **αF**  **αF**  **αG**  **αH**  **αI**  **αL**  **B**  **ERK2**  **A** |
| --- |
| **Figure S1. HDXMS coverage maps for the RSK2:ERK2 complex.** (A) Coverage map of ERK2 alone (top) and ERK2 in the RSK2:ERK2 complex (bottom). (B) Coverage map of RSK2 in the RSK2:ERK2 complex. All coverage maps occur at 2 minutes of exchange, and secondary structure elements are indicated above the sequences. |

| 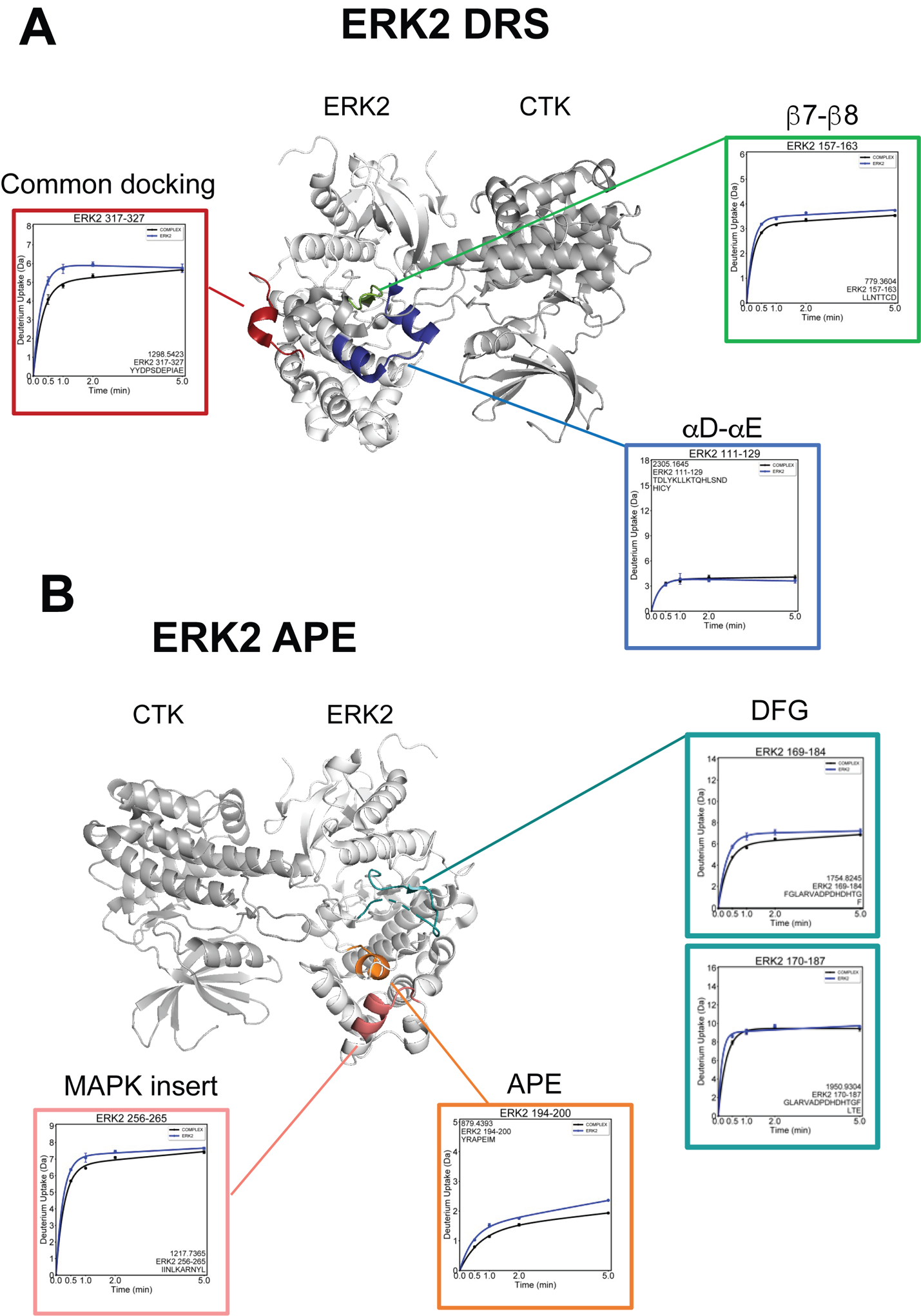 |
| --- |
| **Figure S2:** **Changes in ERK2 HDXMS profile upon RSK2 binding in the context of CTK:ERK2.** (A) ERK2 uptake plots at its DRS with and without RSK2. (B) Uptake plots of additional ERK2 peptides with changes in uptake upon RSK2 binding |
|  |

| 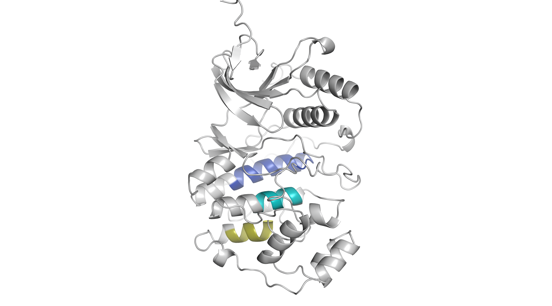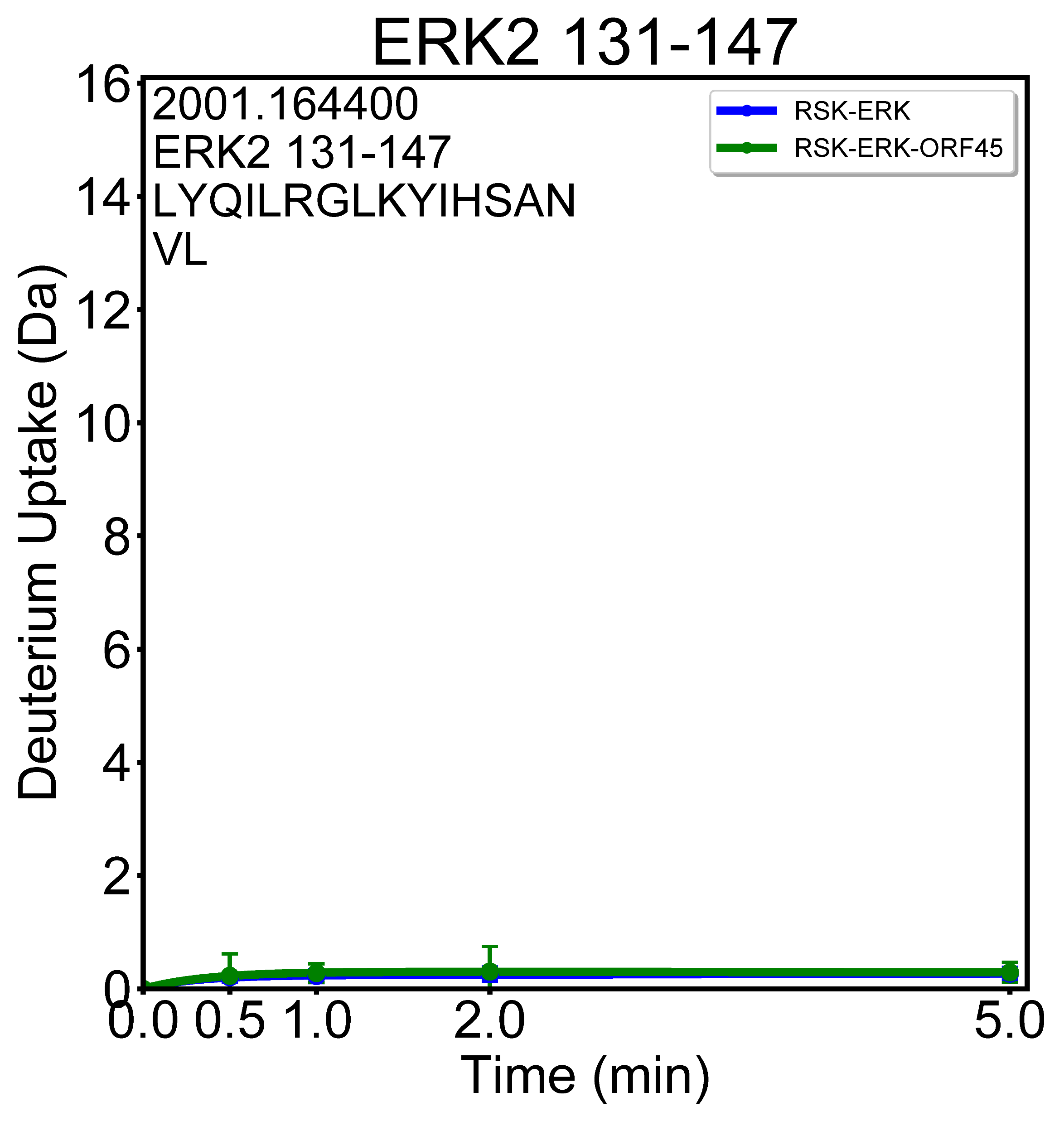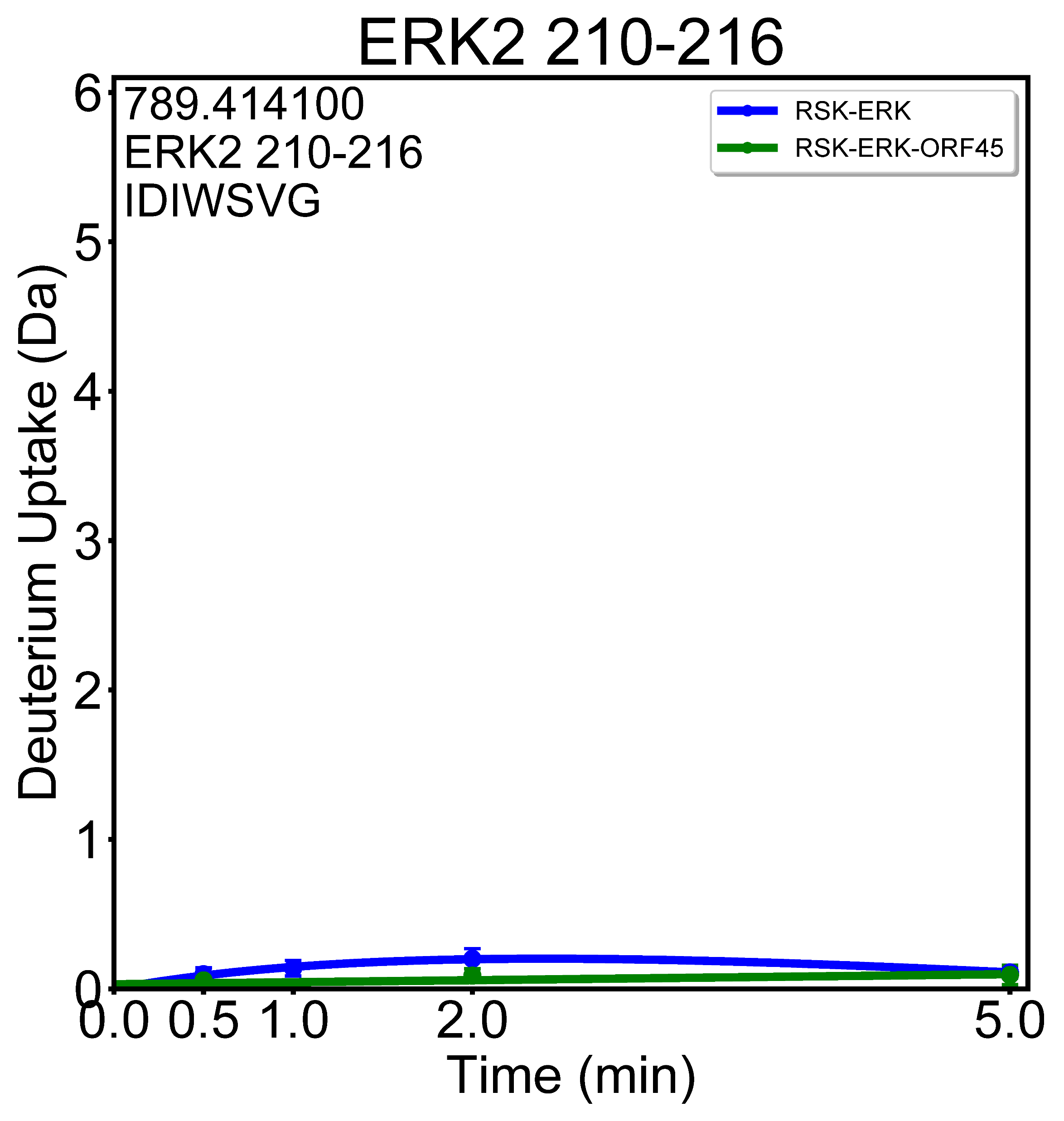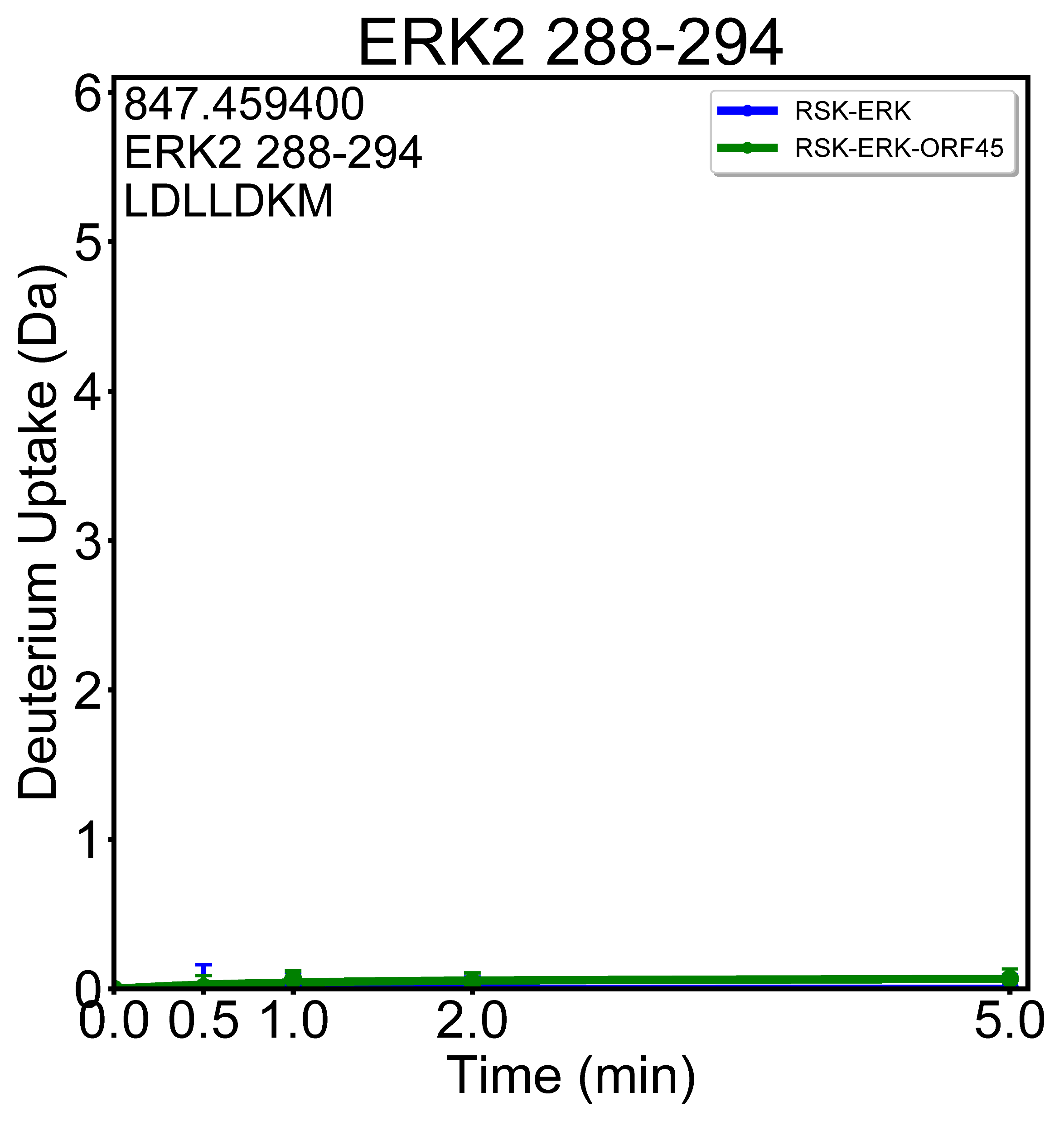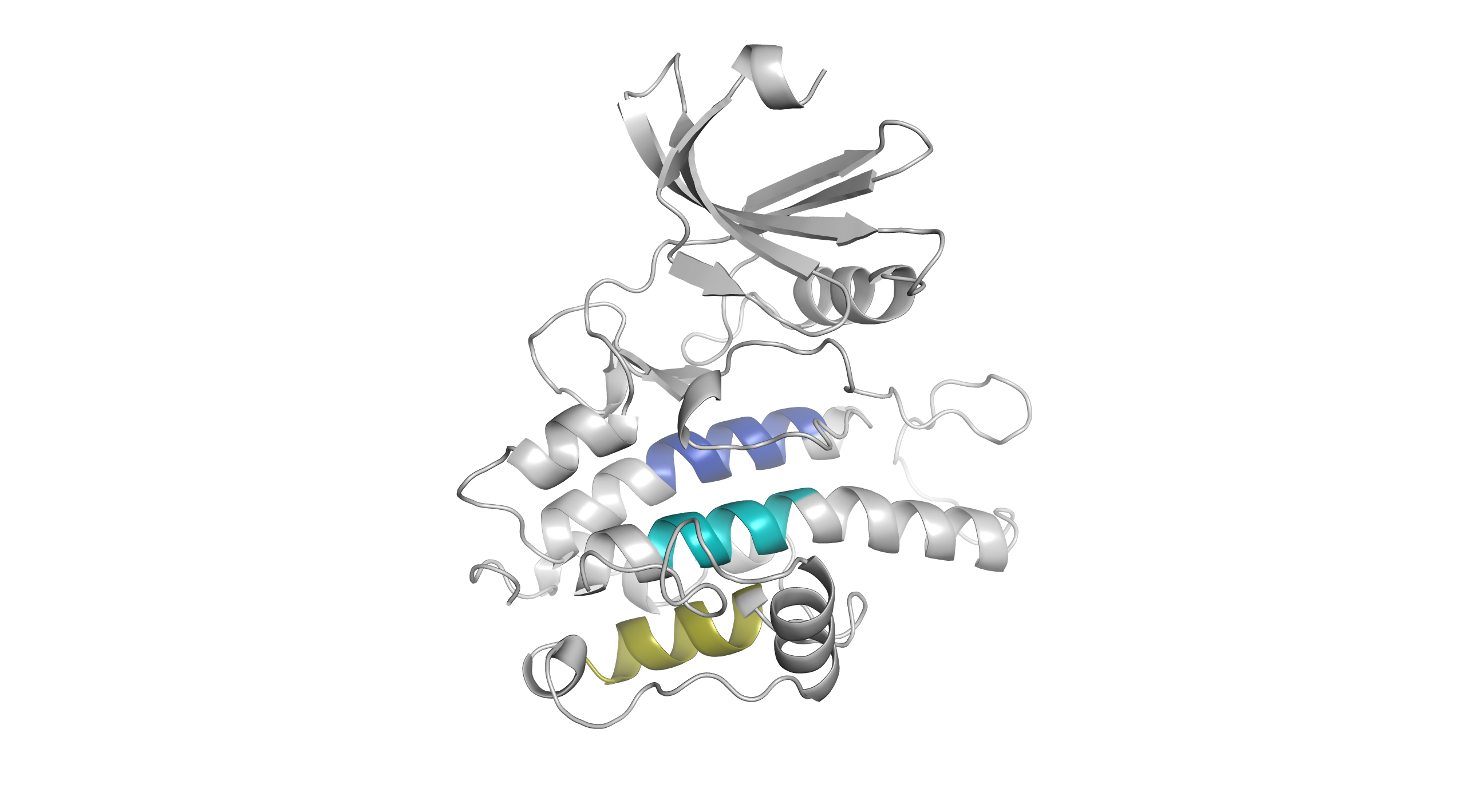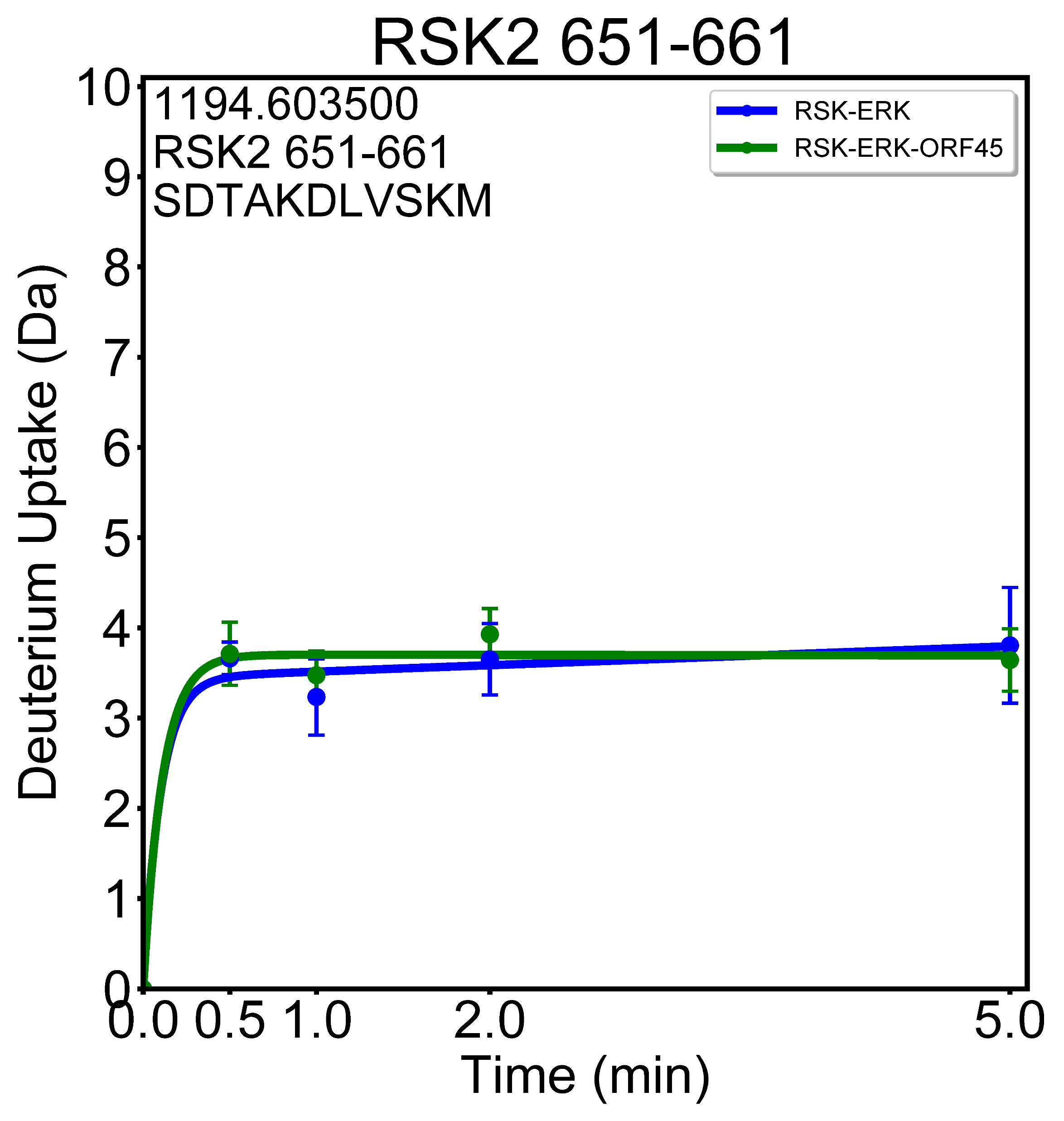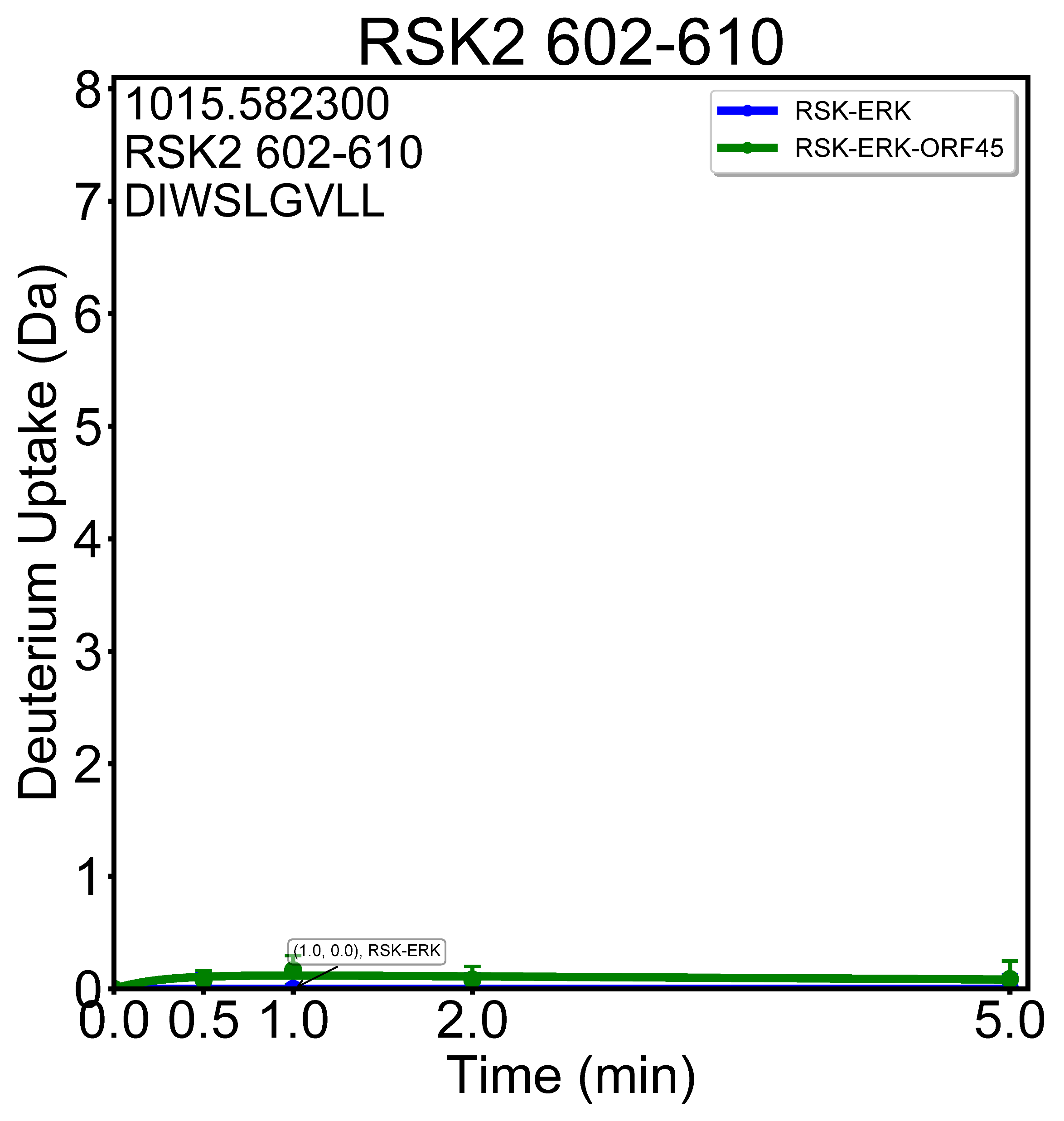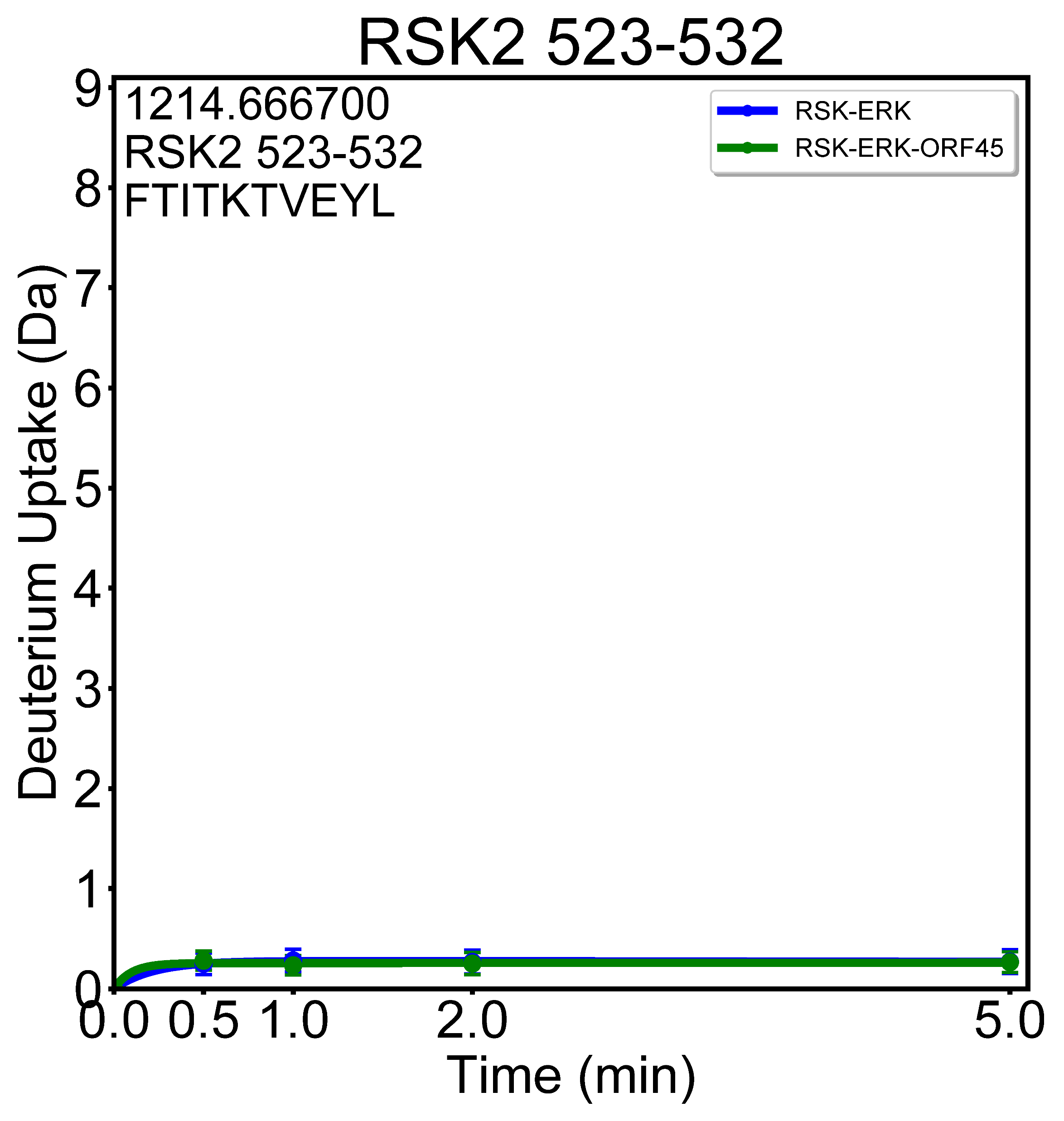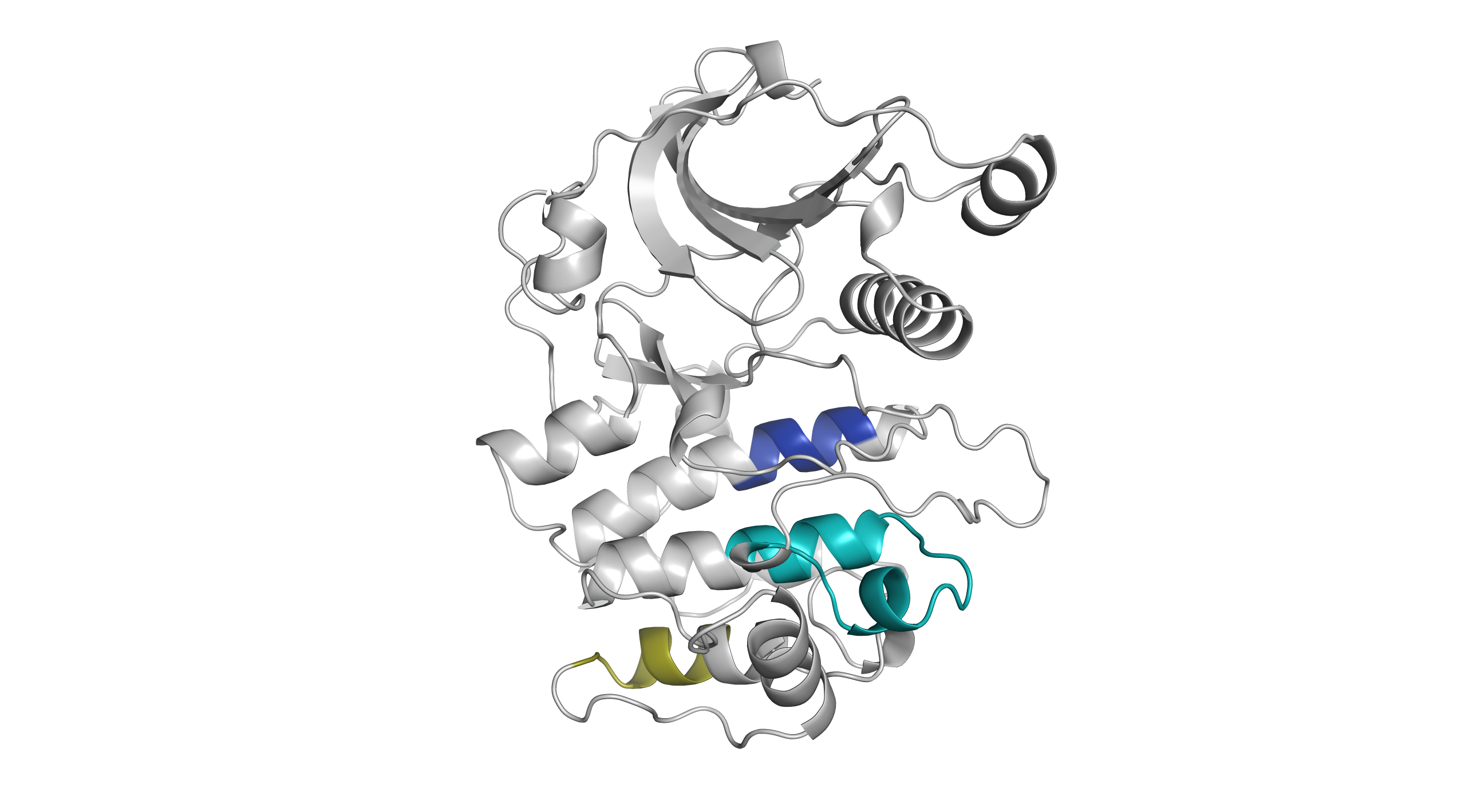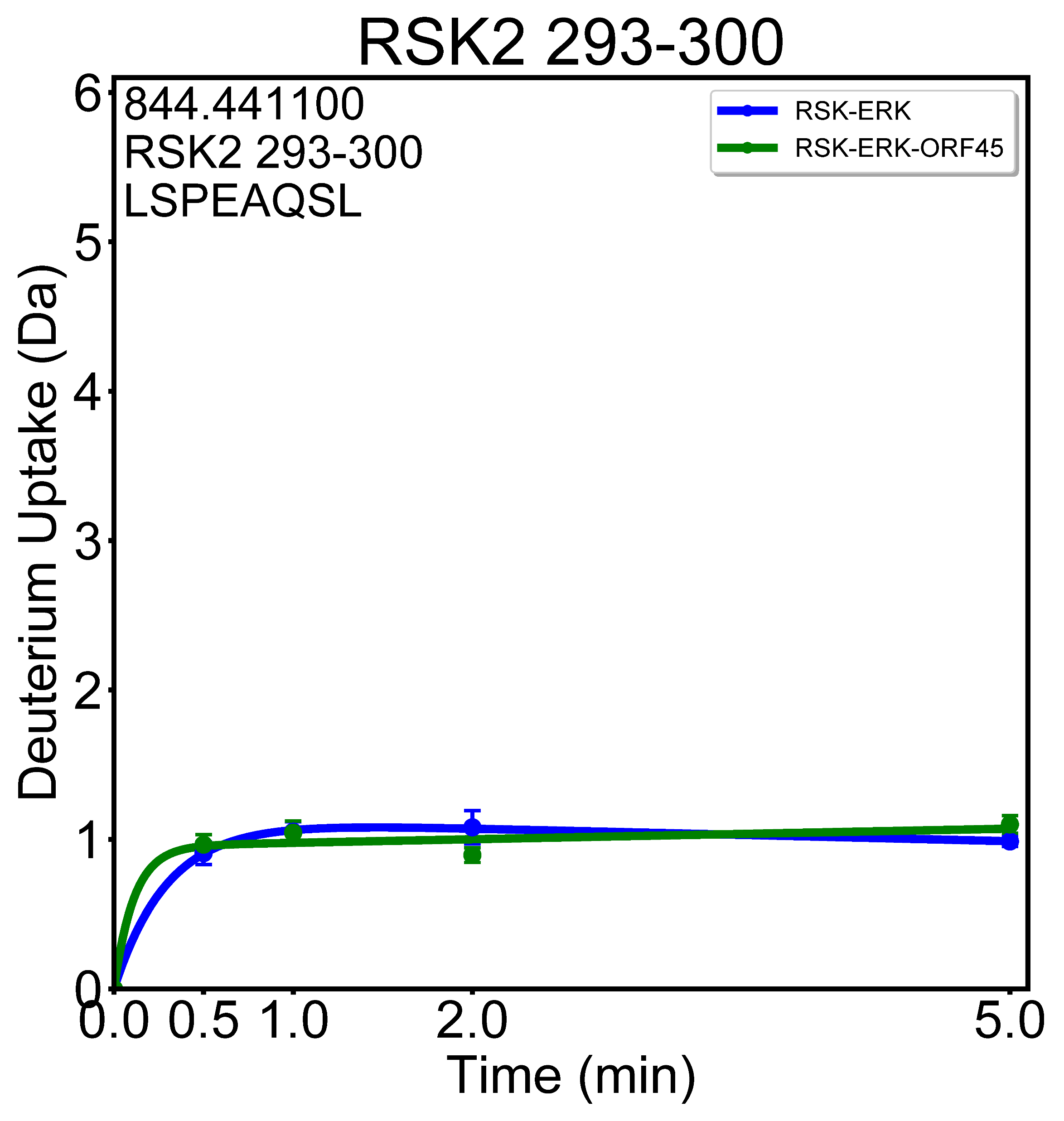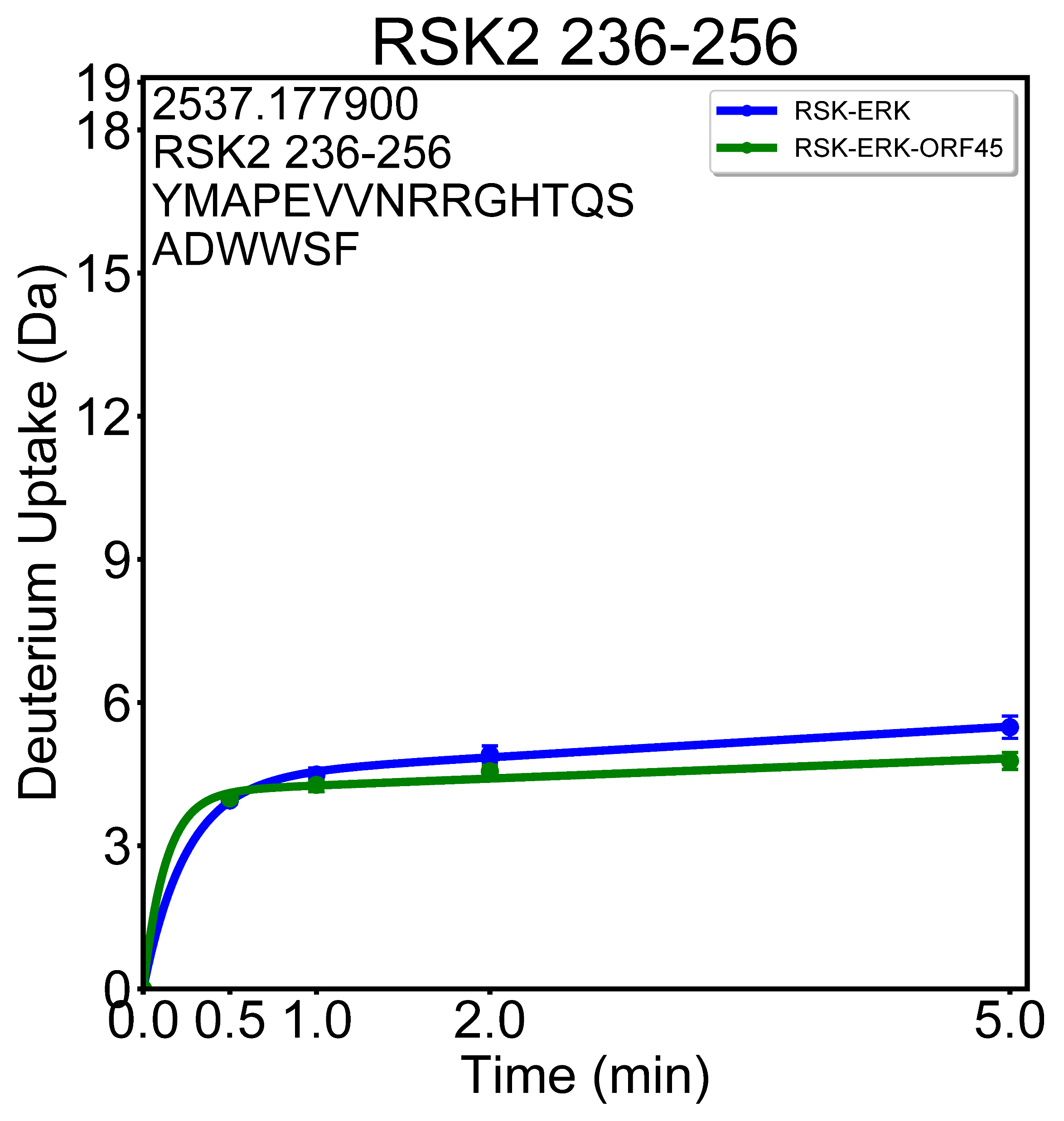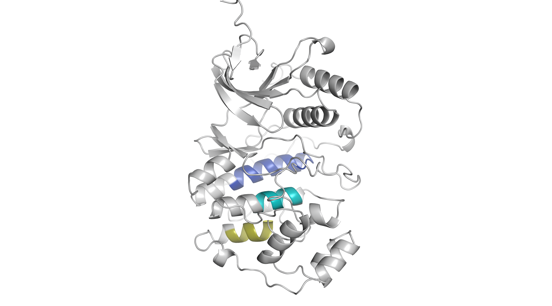 **ERK2**  αE  αH  αF  αH  αF  αE  αH  αE  αF  **NTK**  **CTK**  **C** 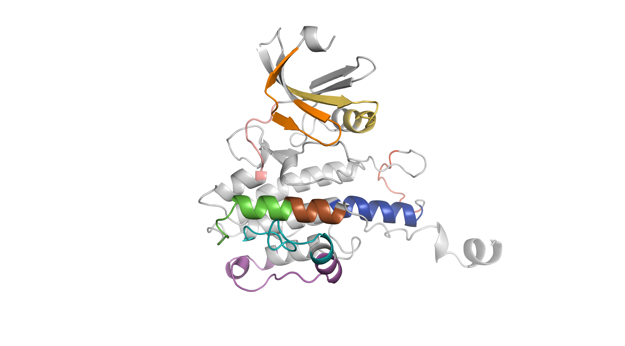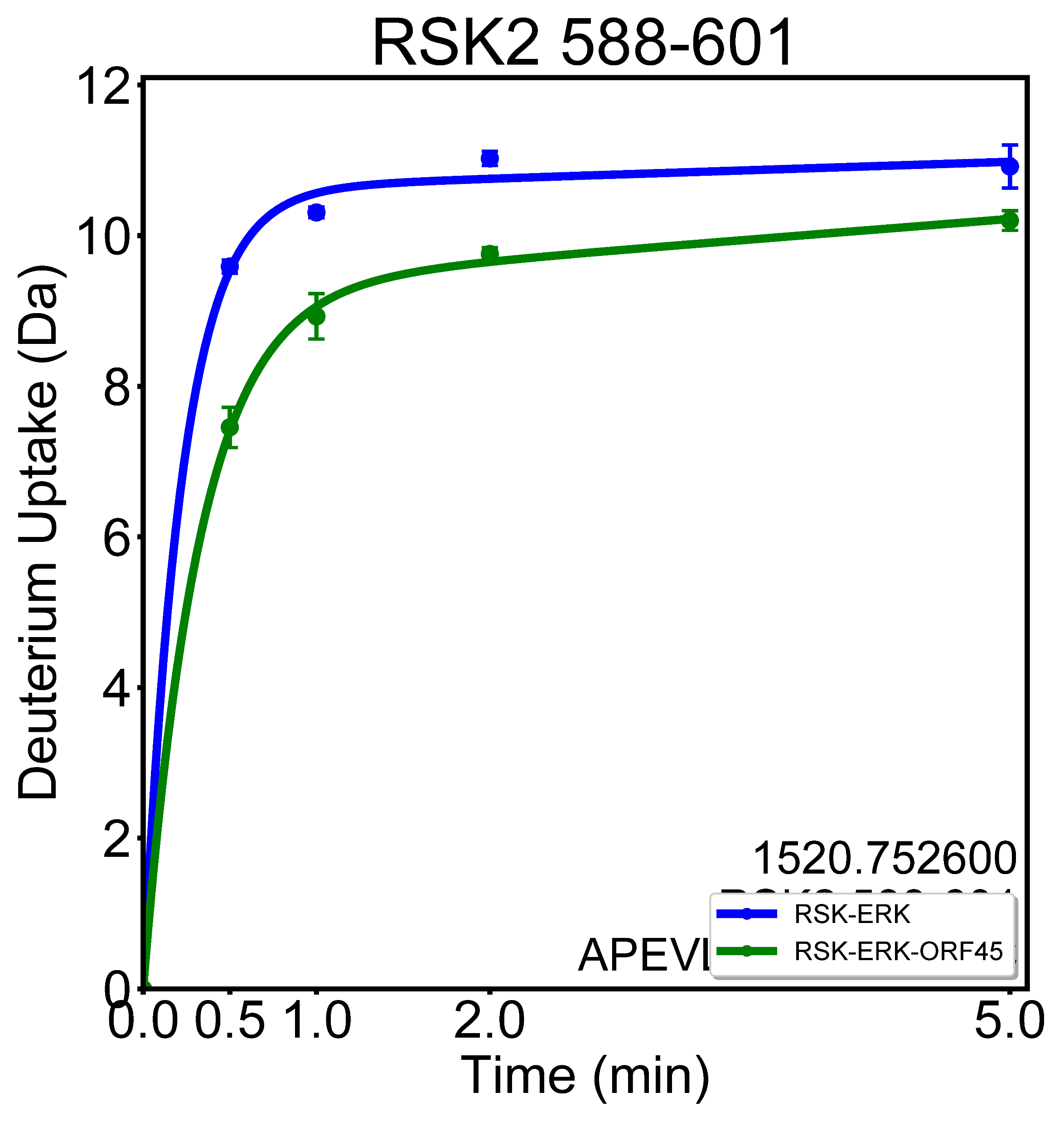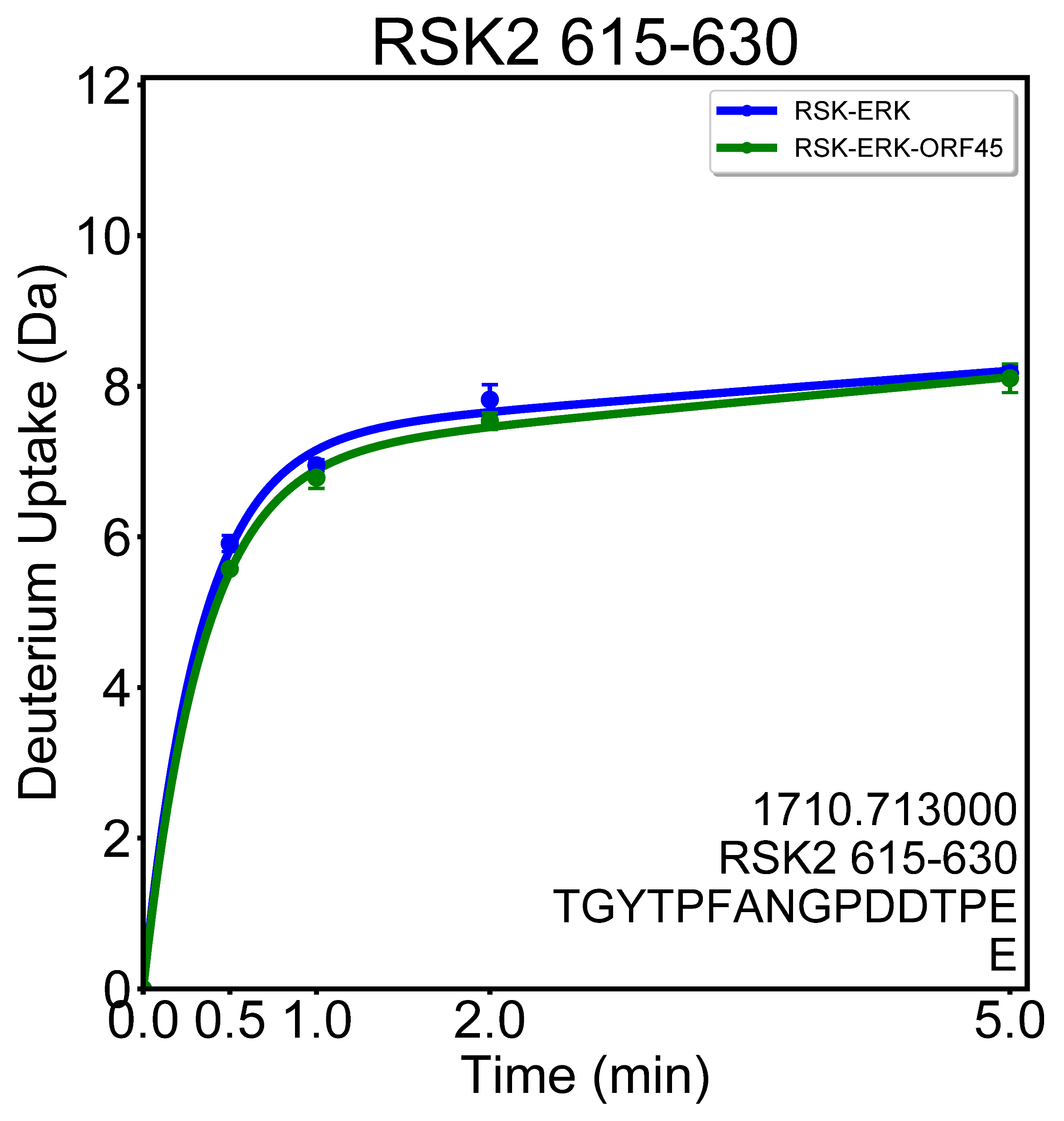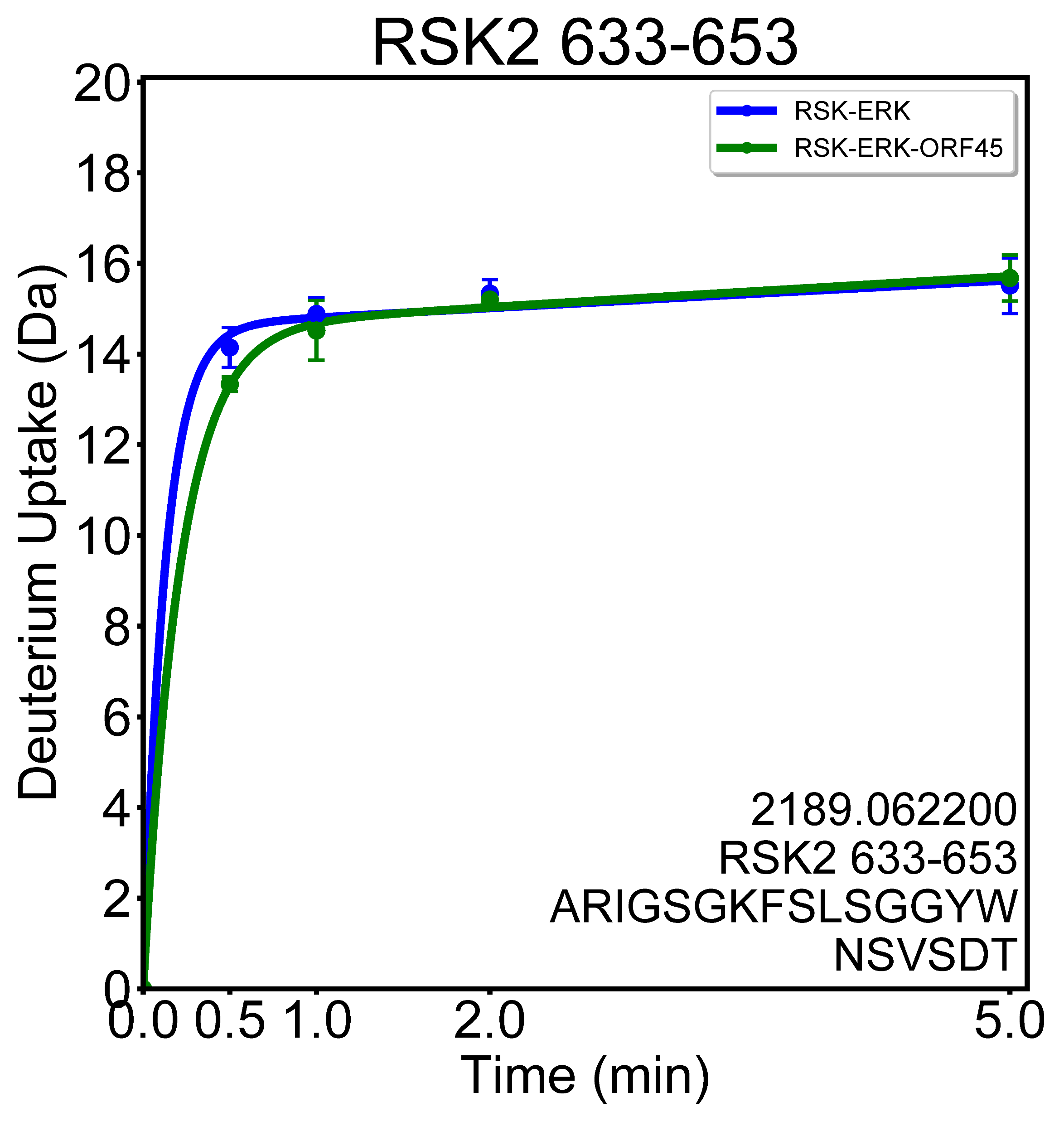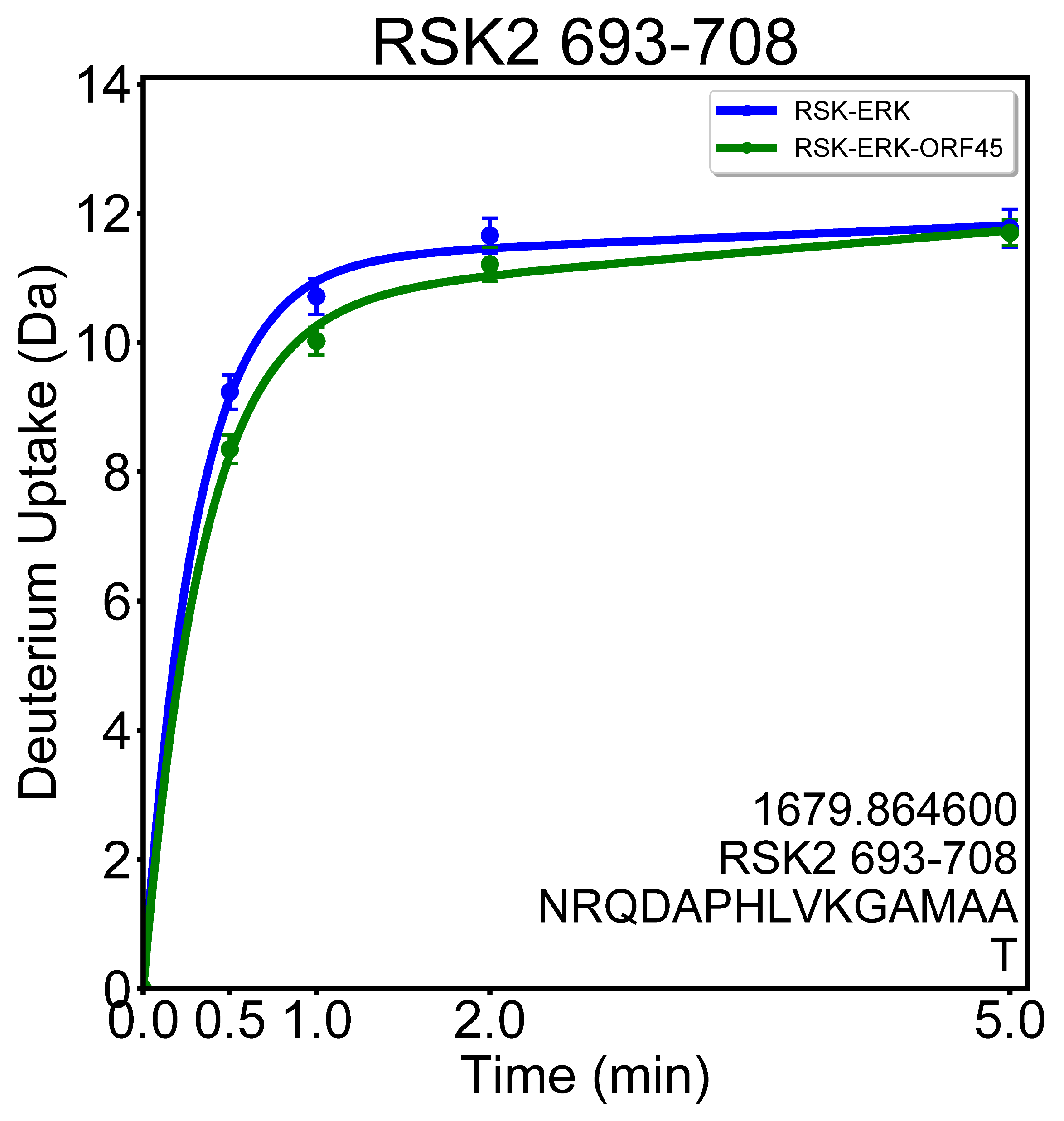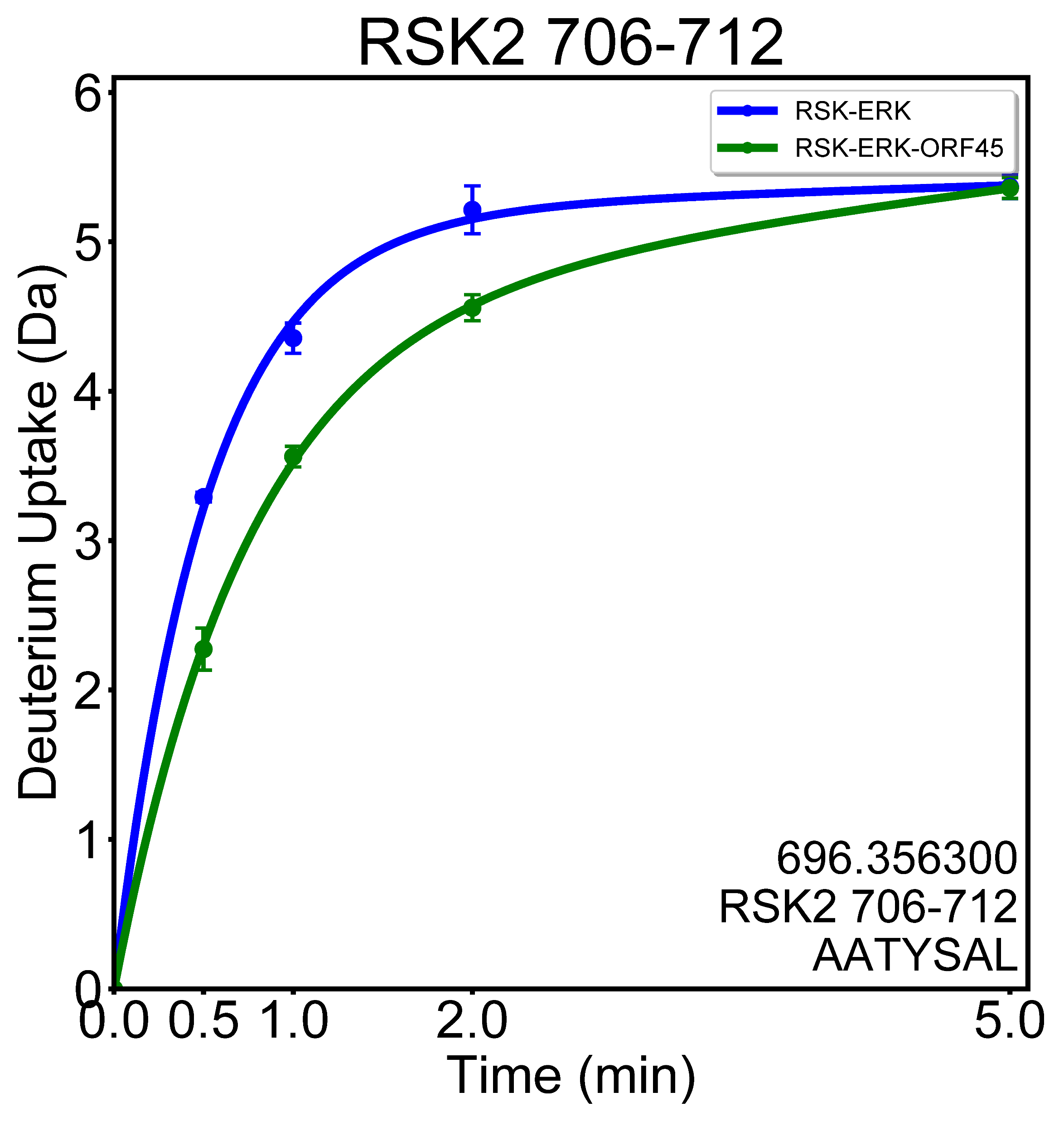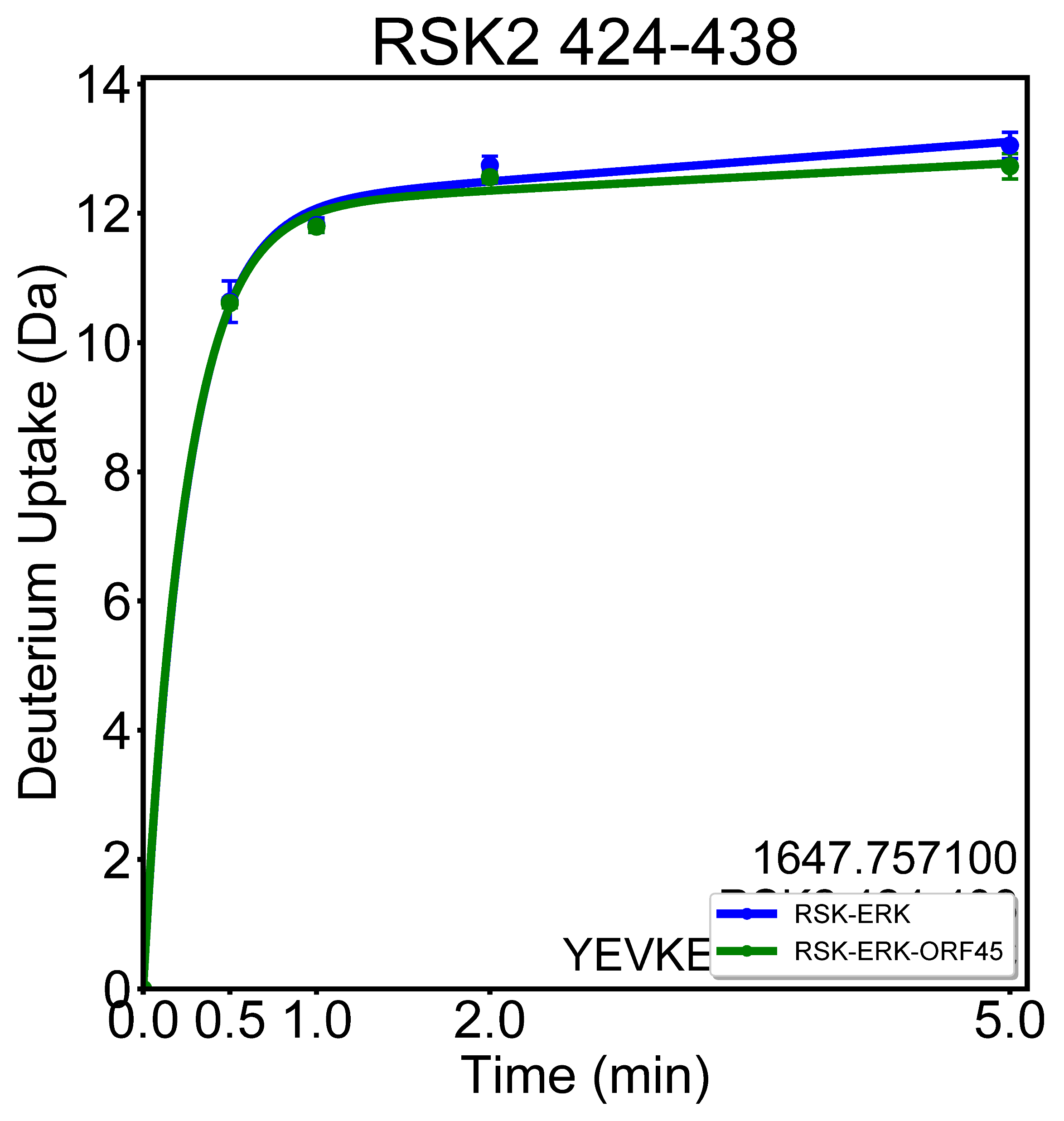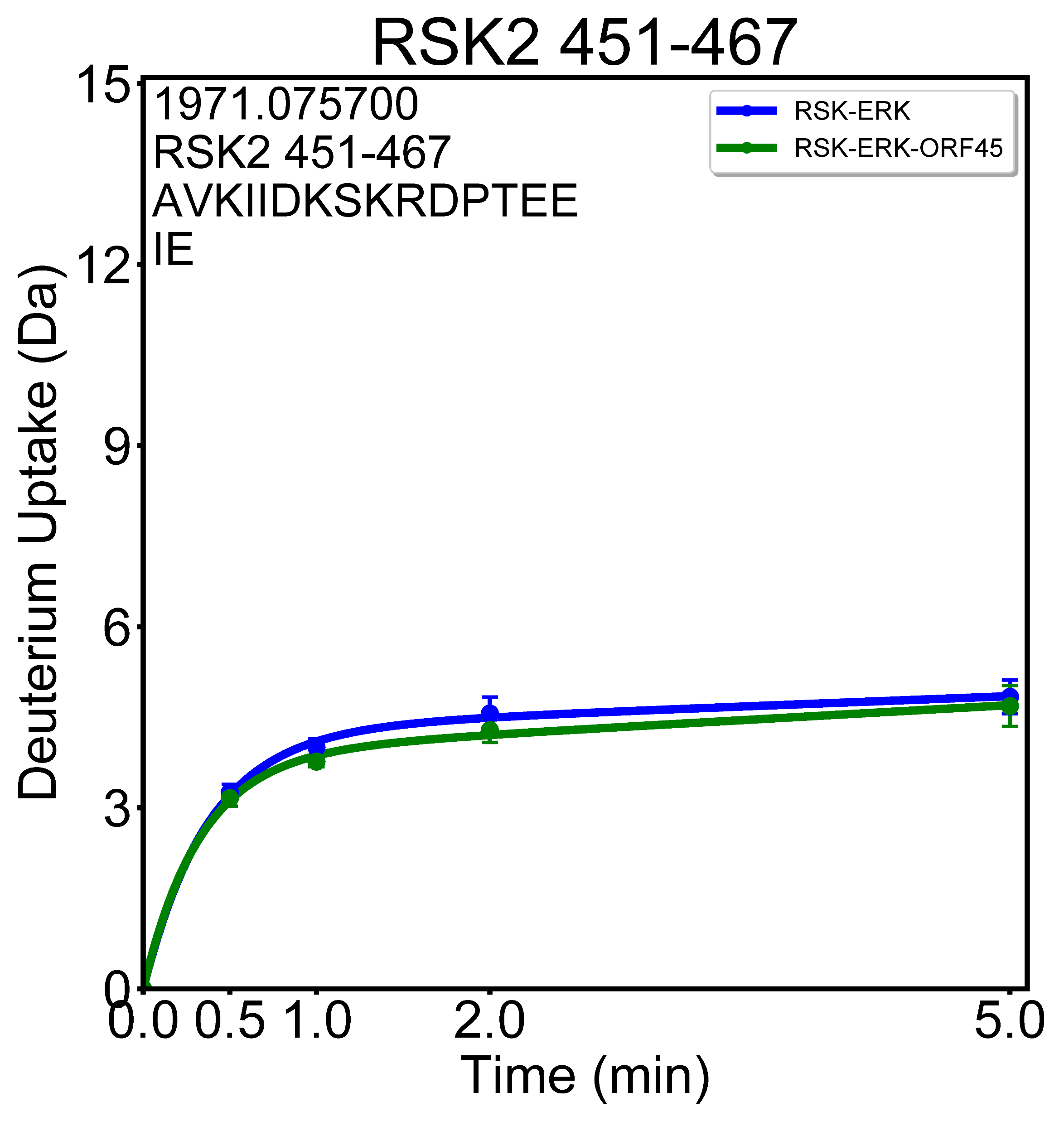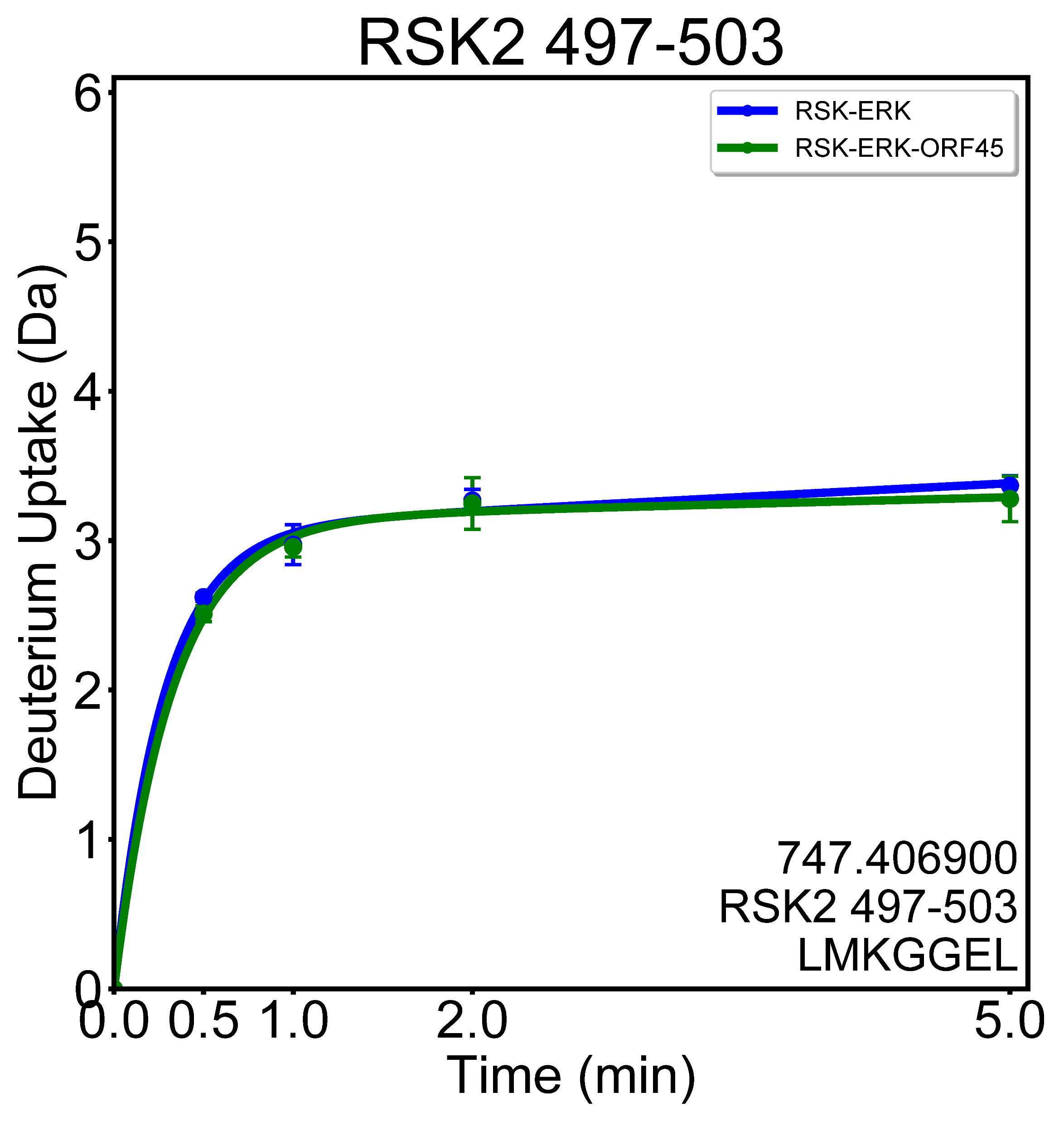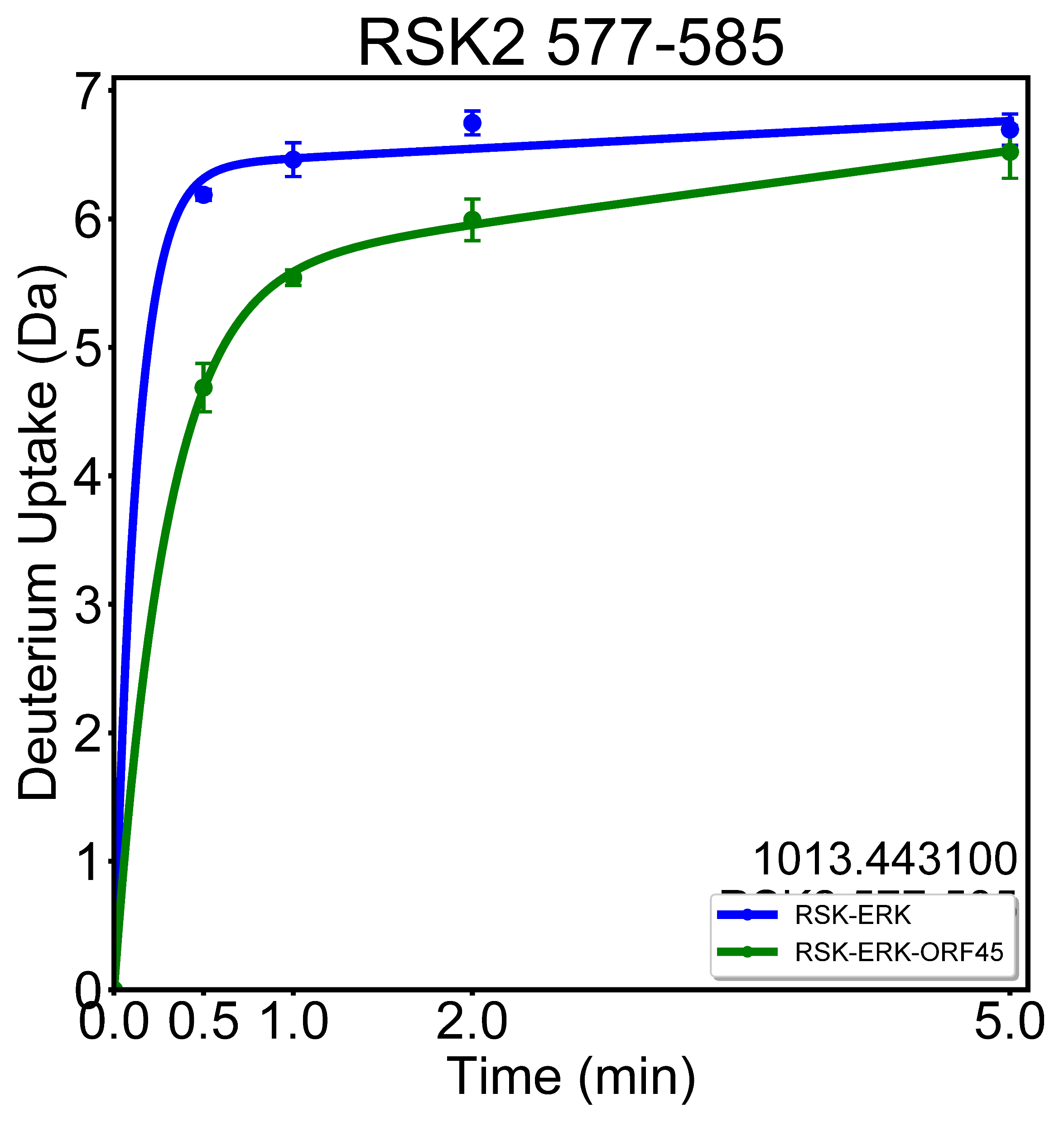 **CTK**  β3-αC  Activation segment  Gly loop  Hinge  αL  αL  APE / extended αF  αF-αG  αG-αH 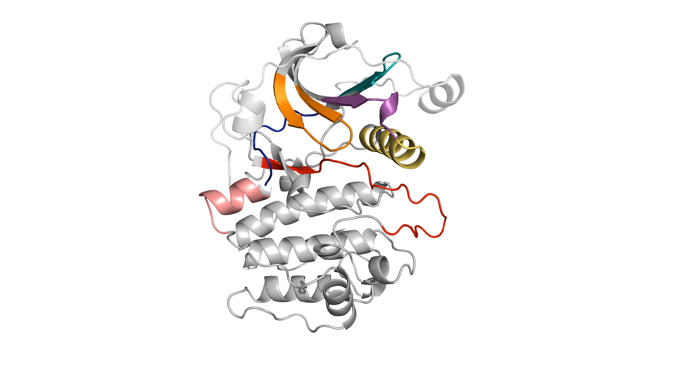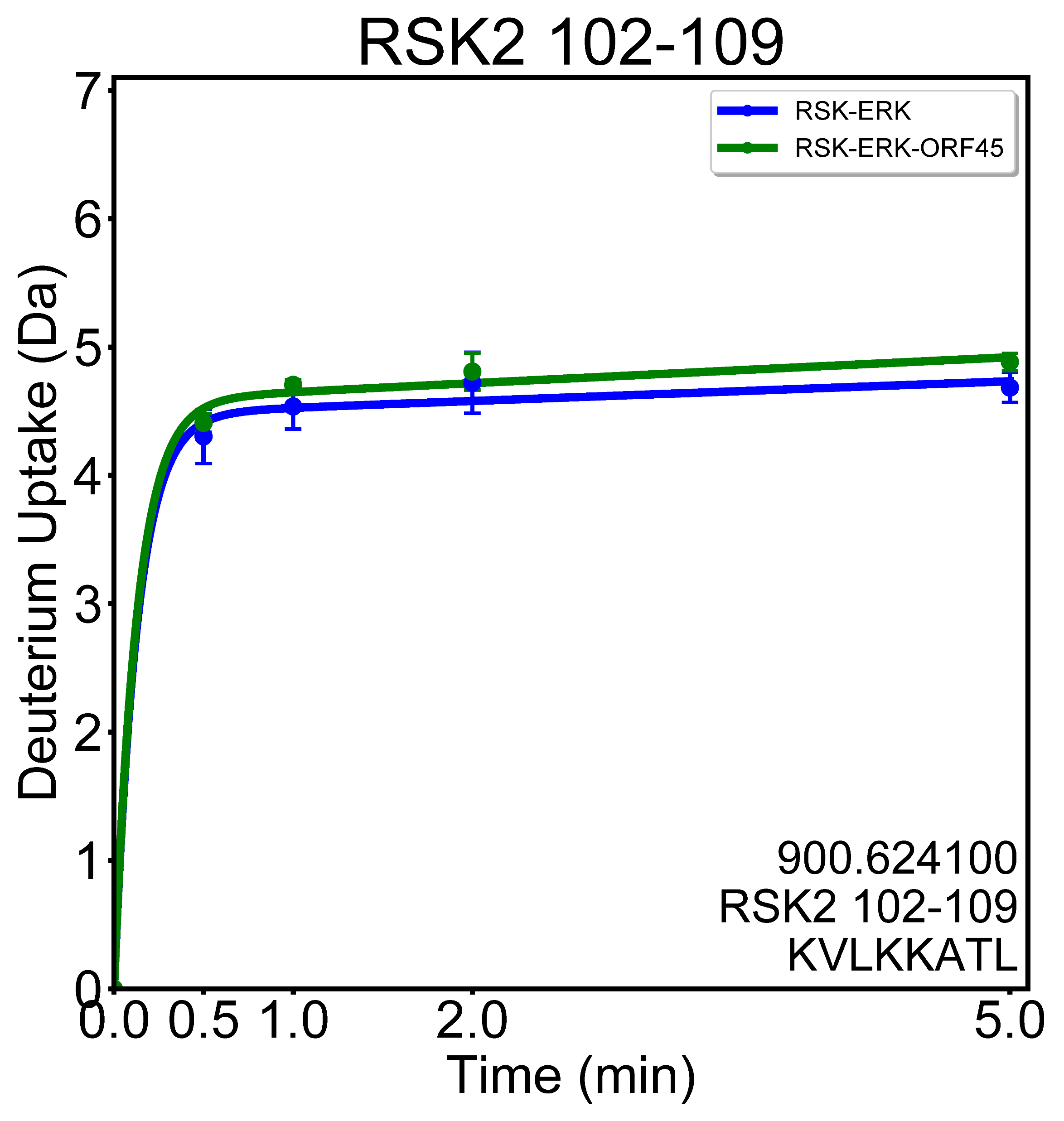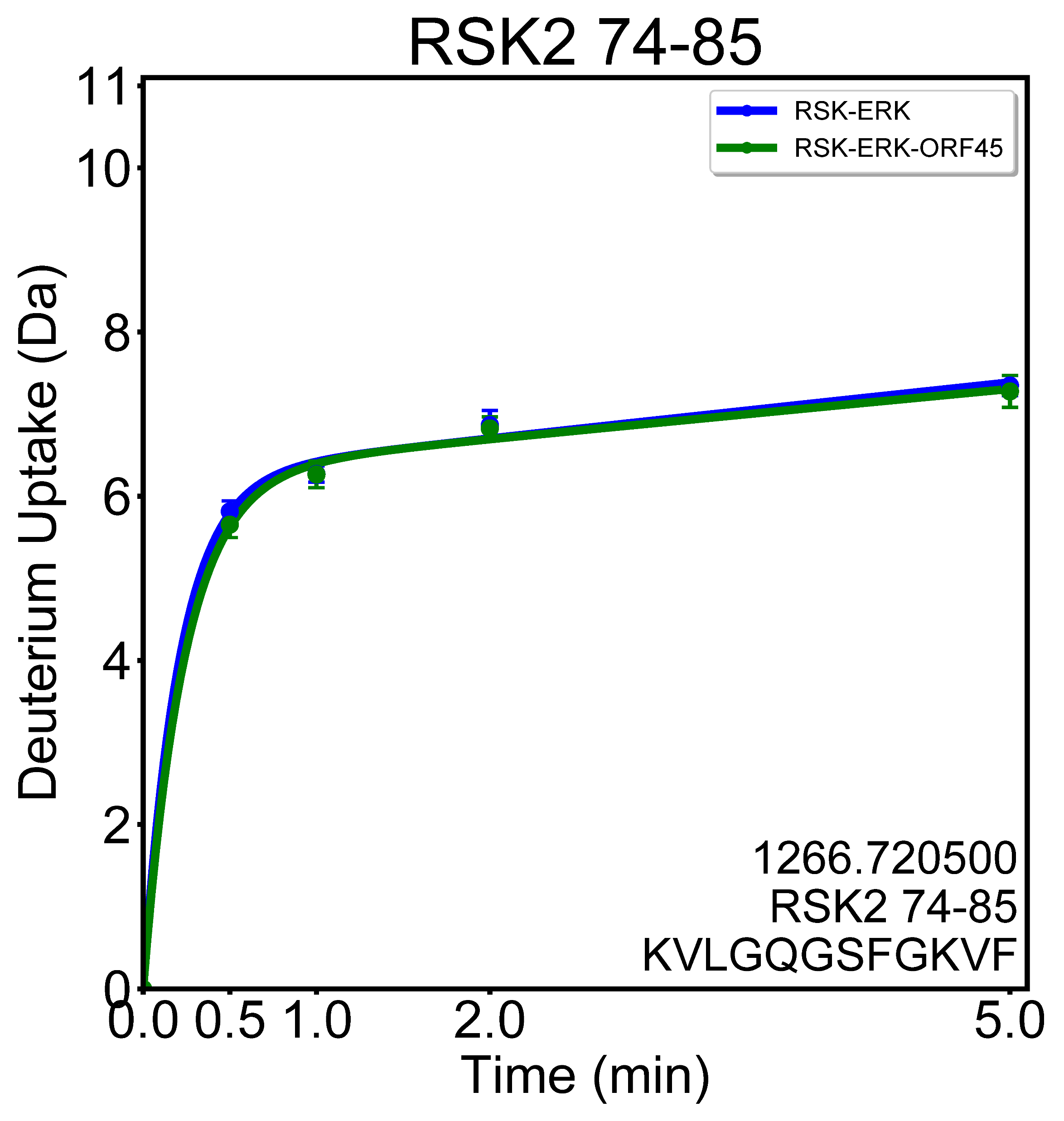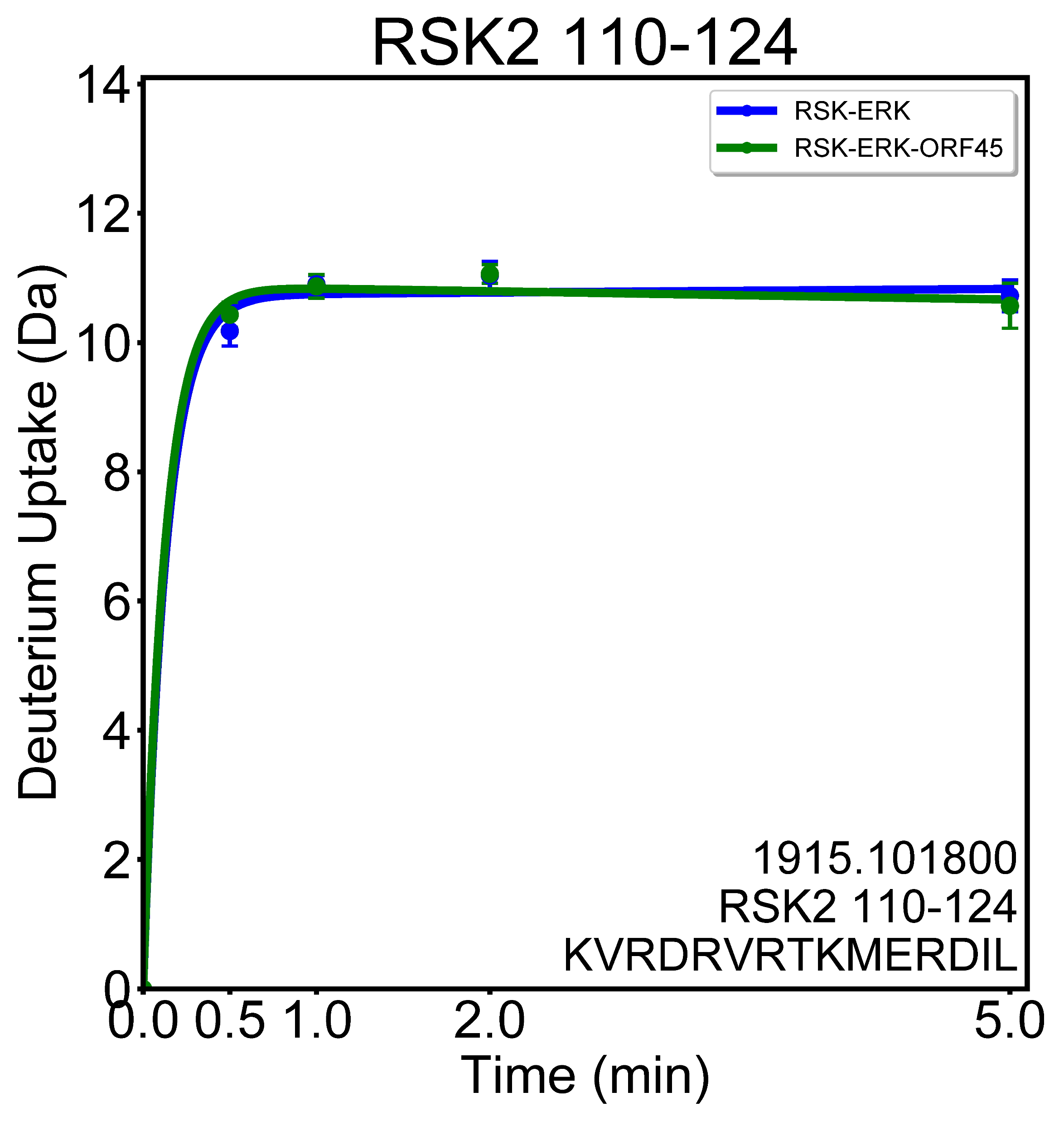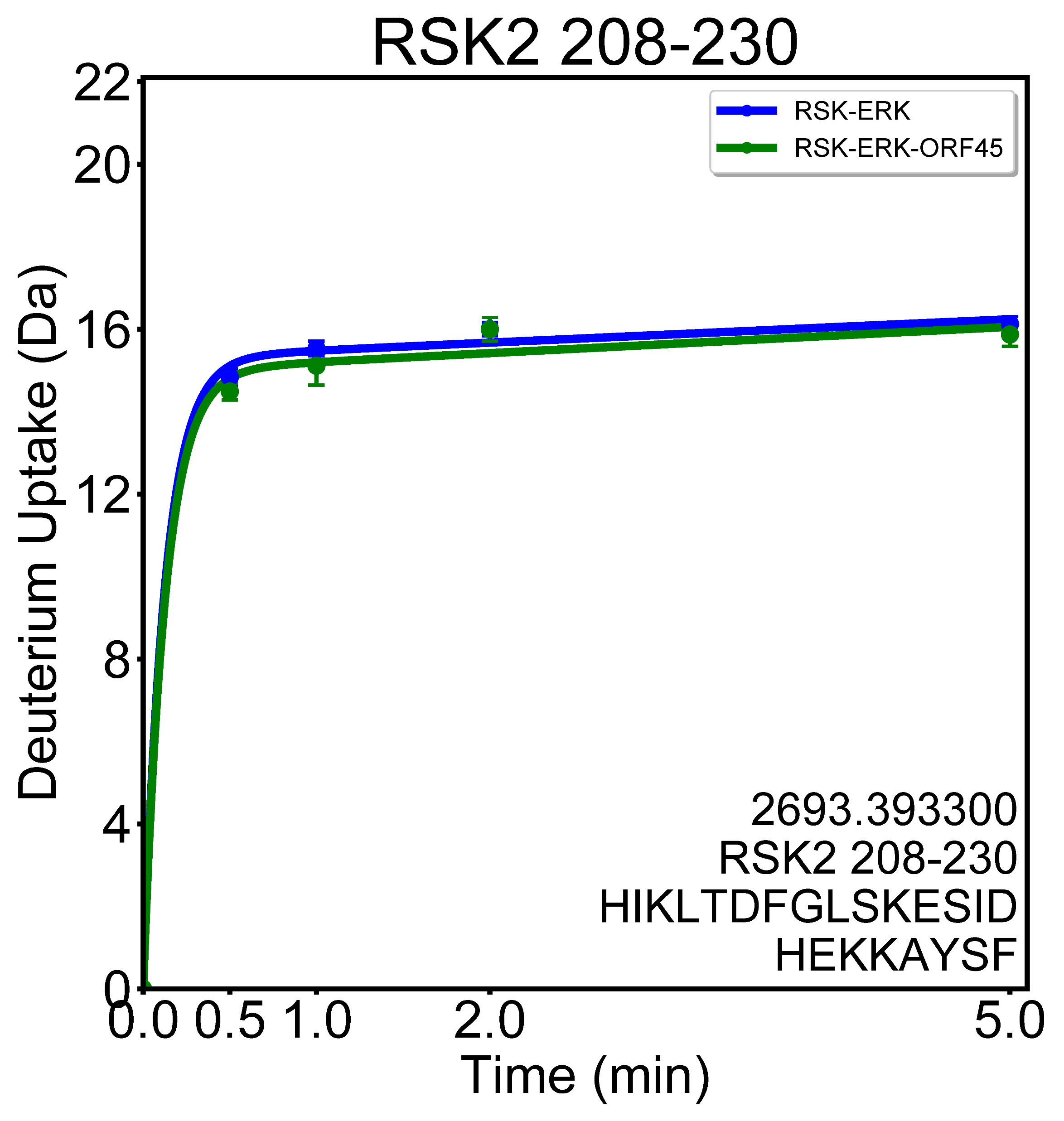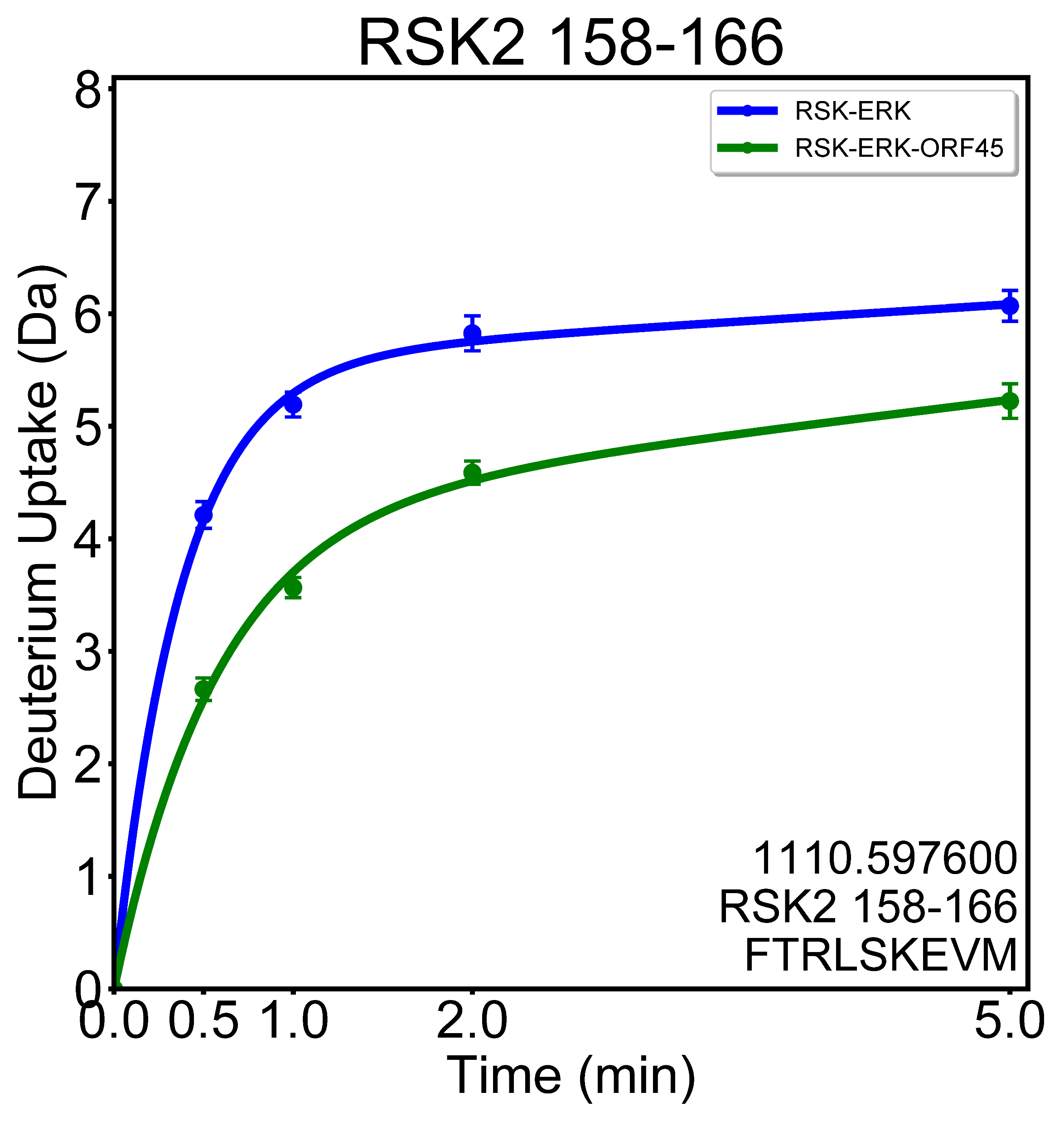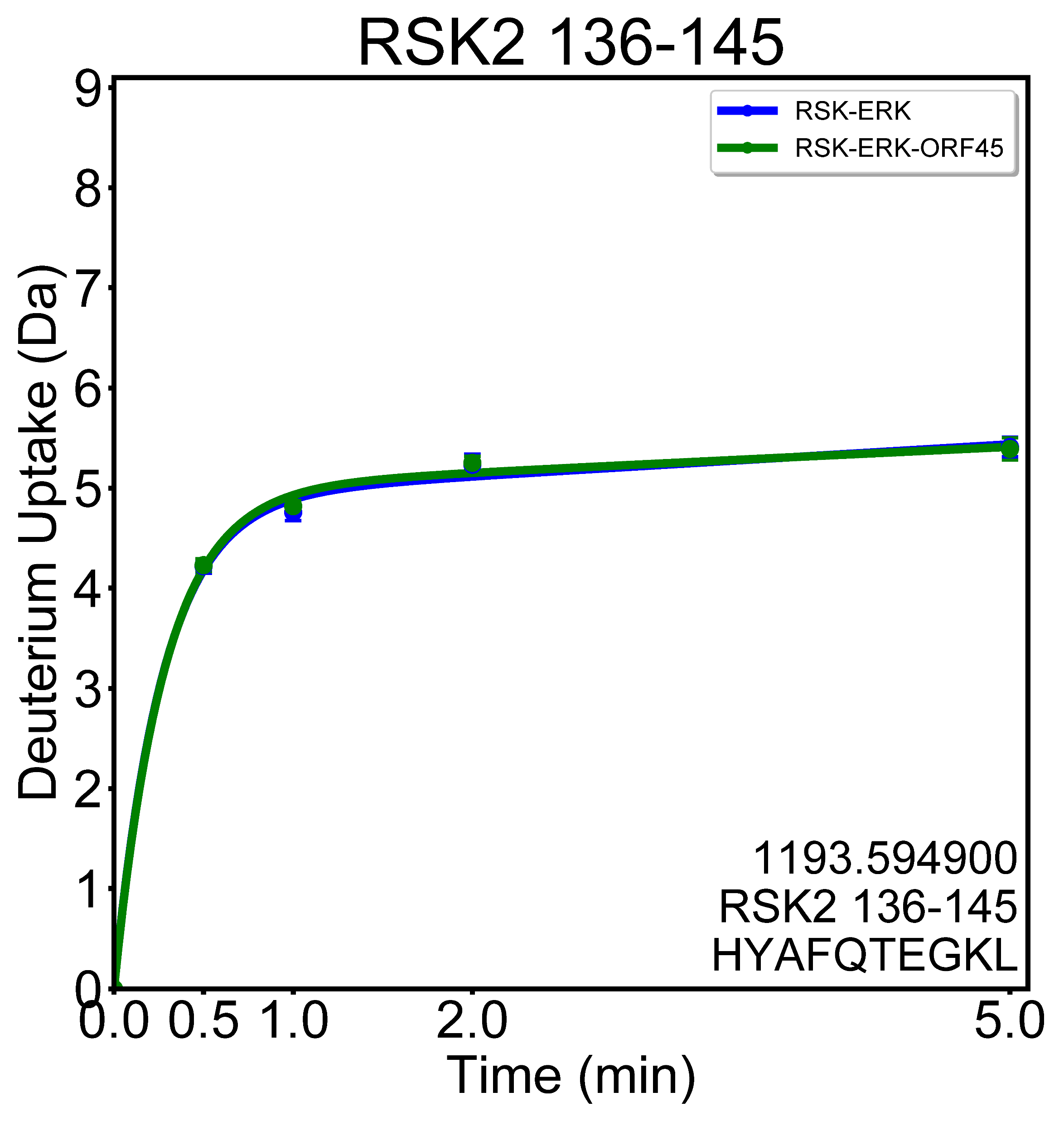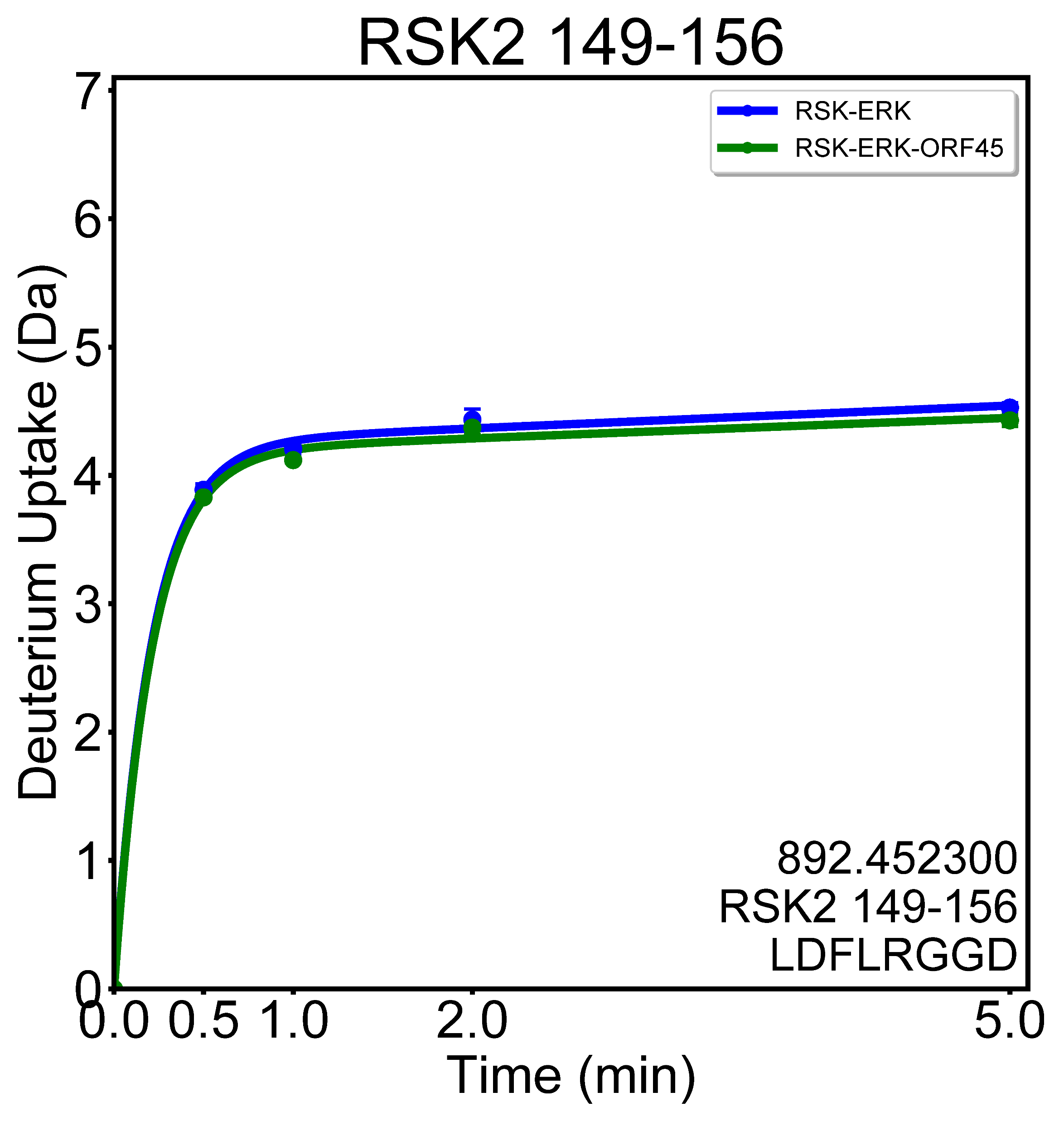 **NTK**  αC  β3-αC  Activation segment  β4-β5  Gly loop  Hinge  αD  **B**  **A**  **D** 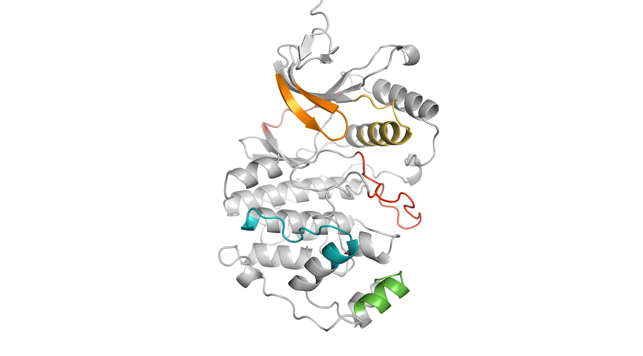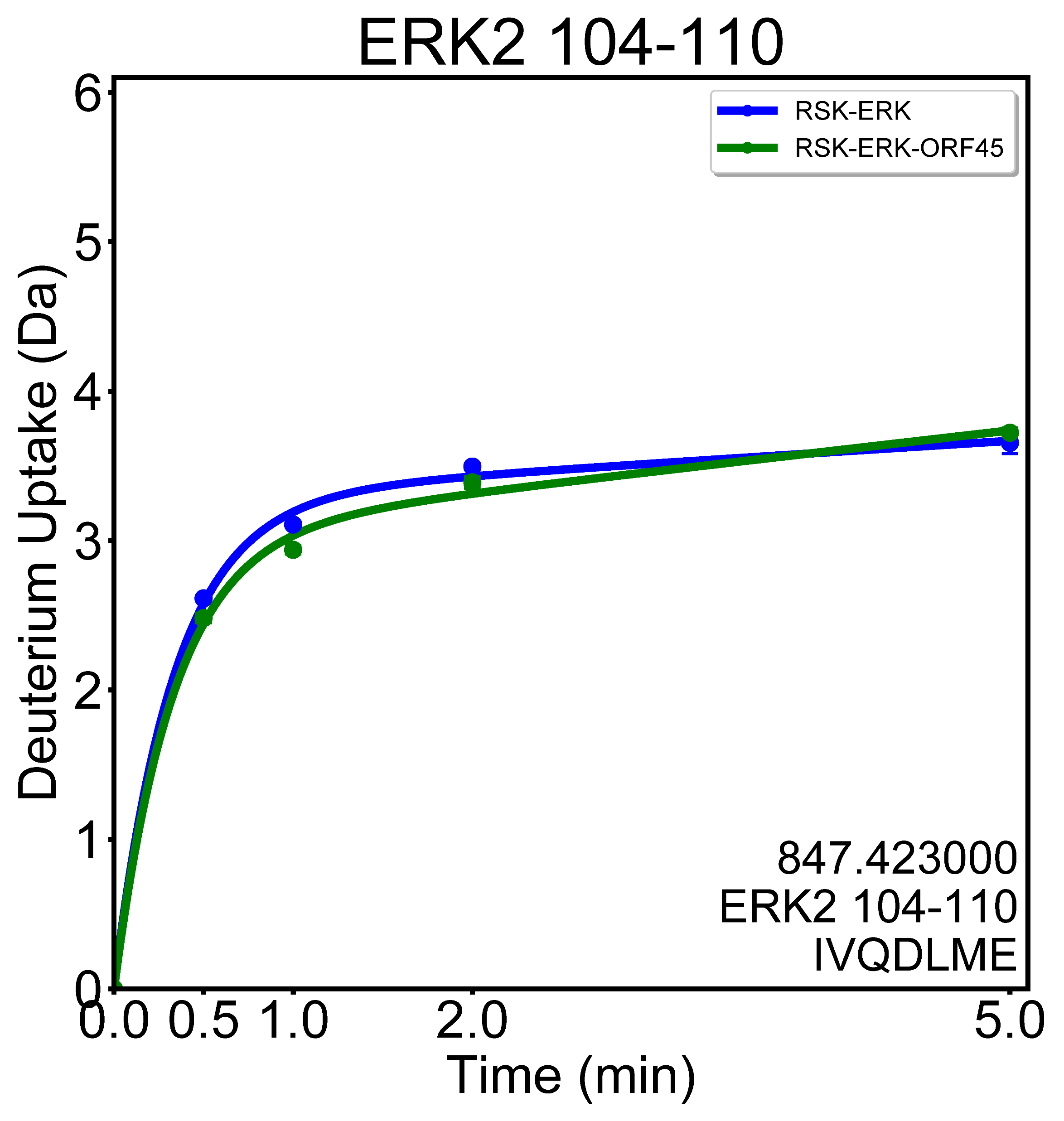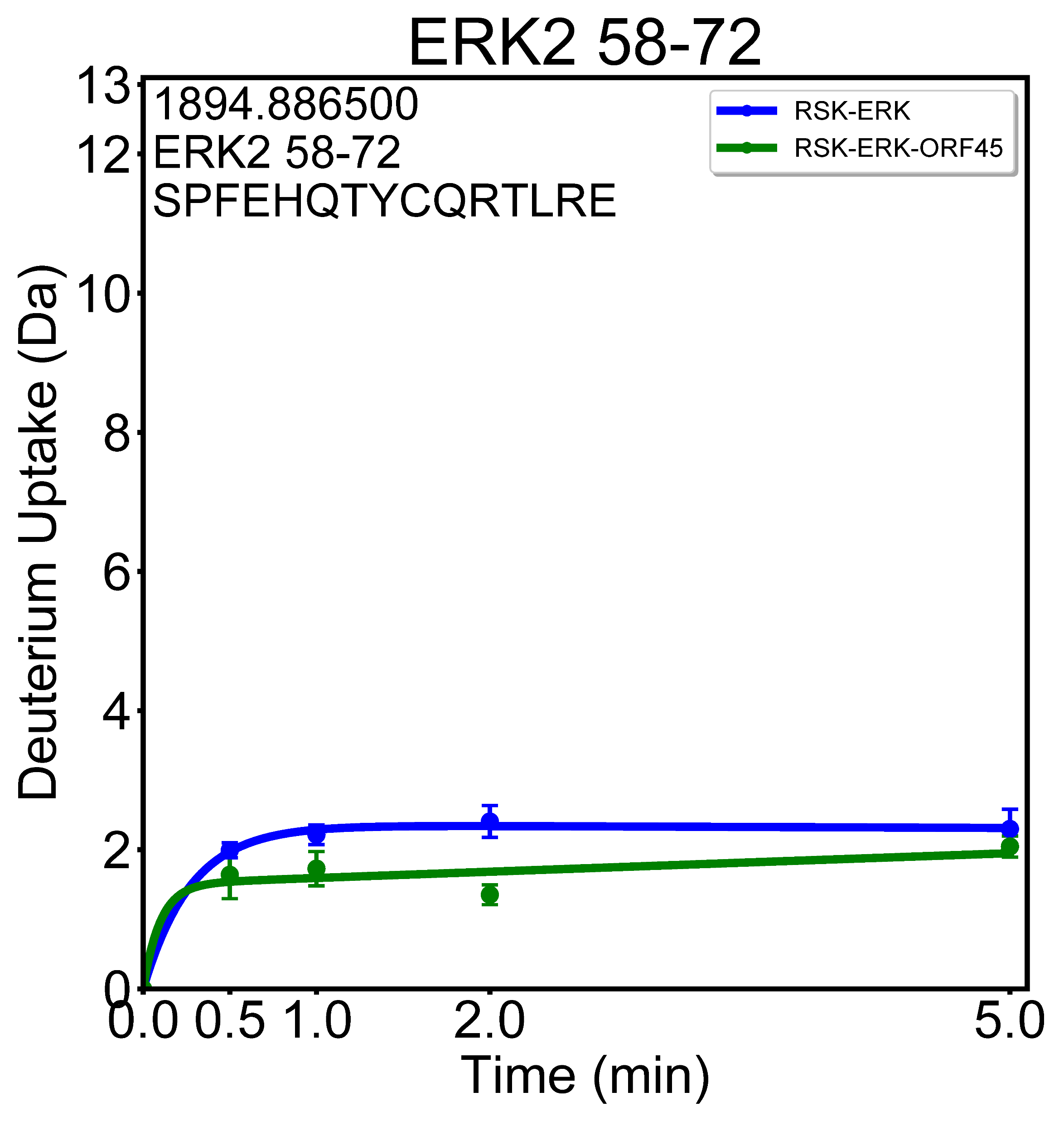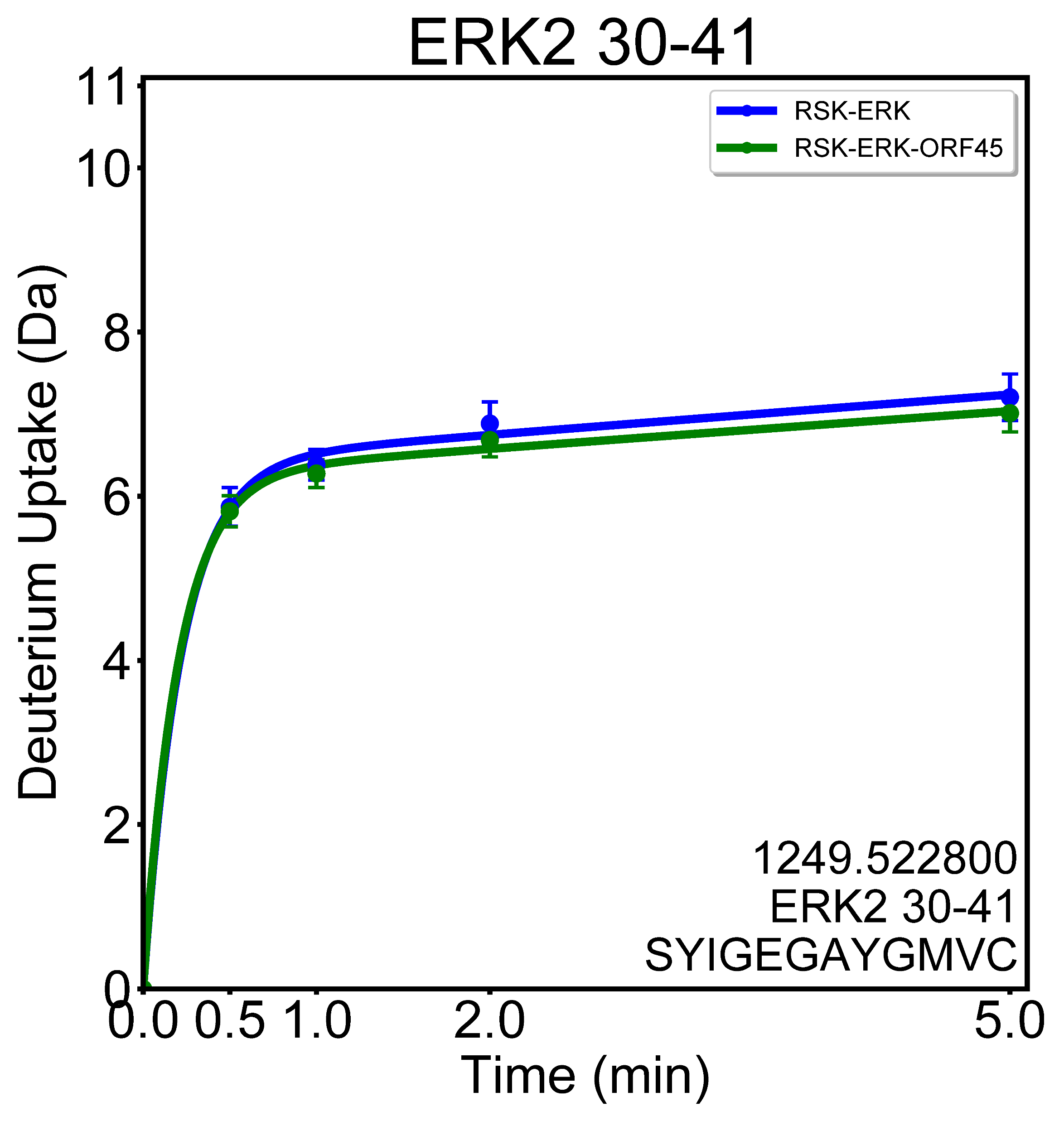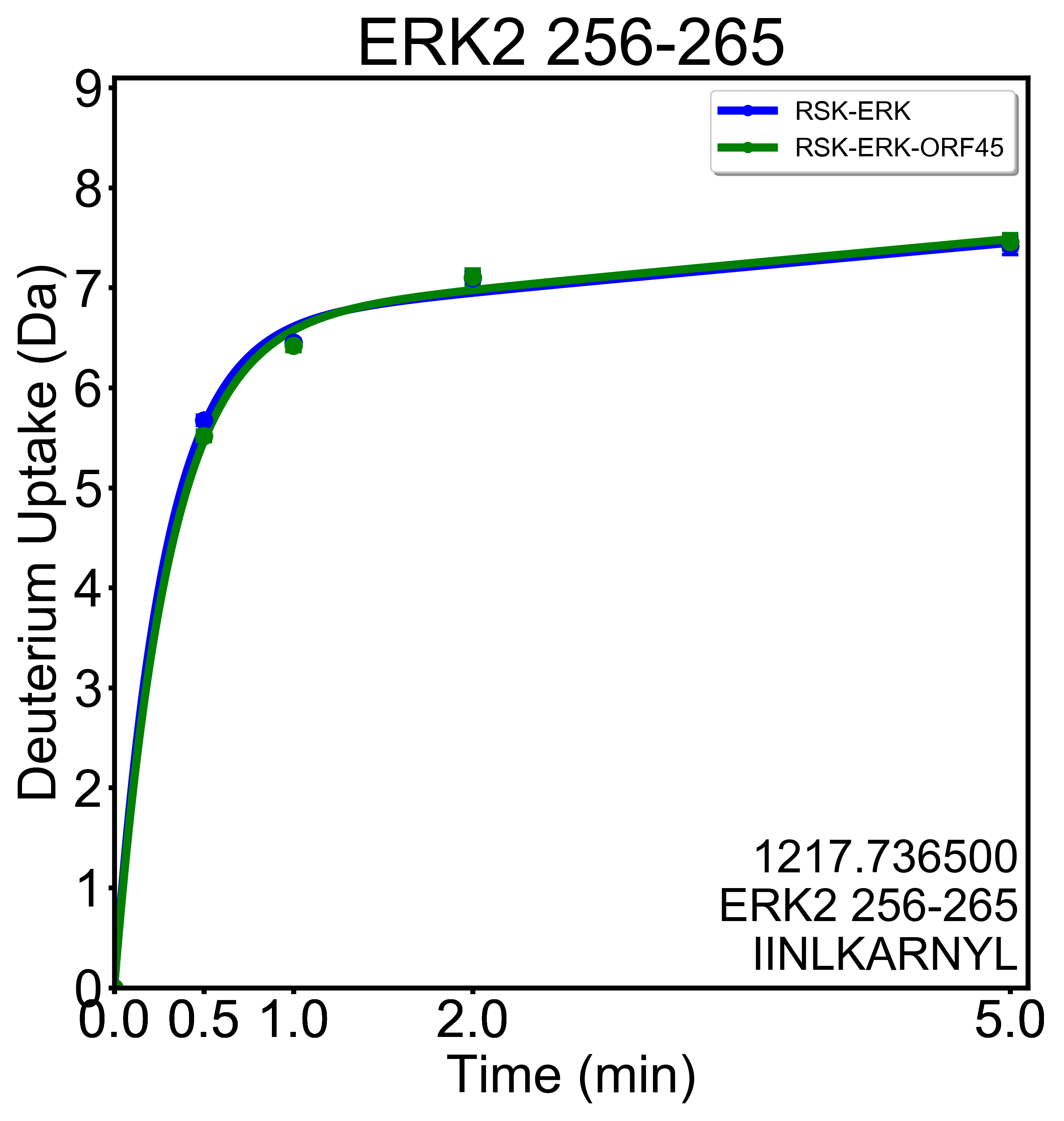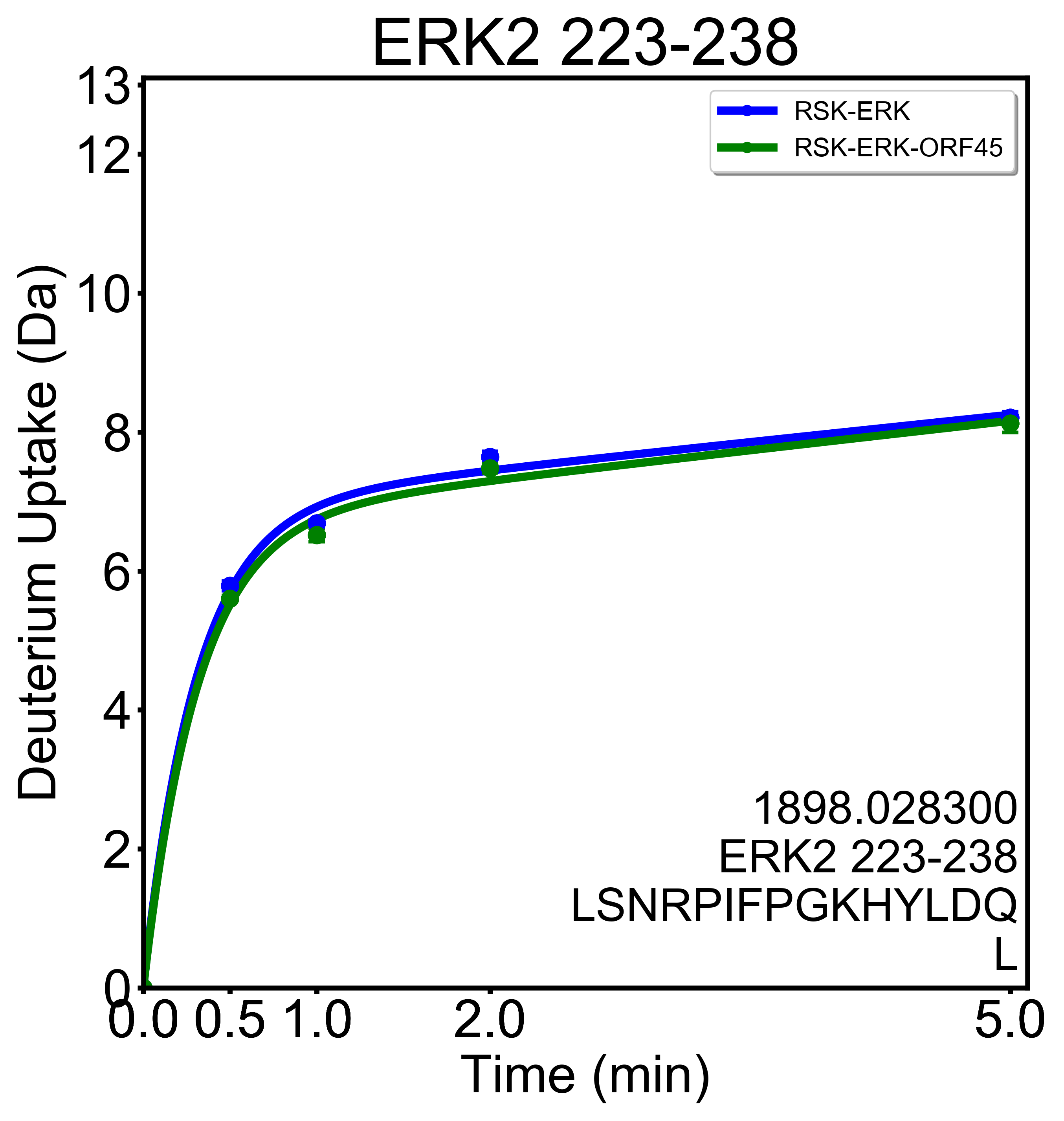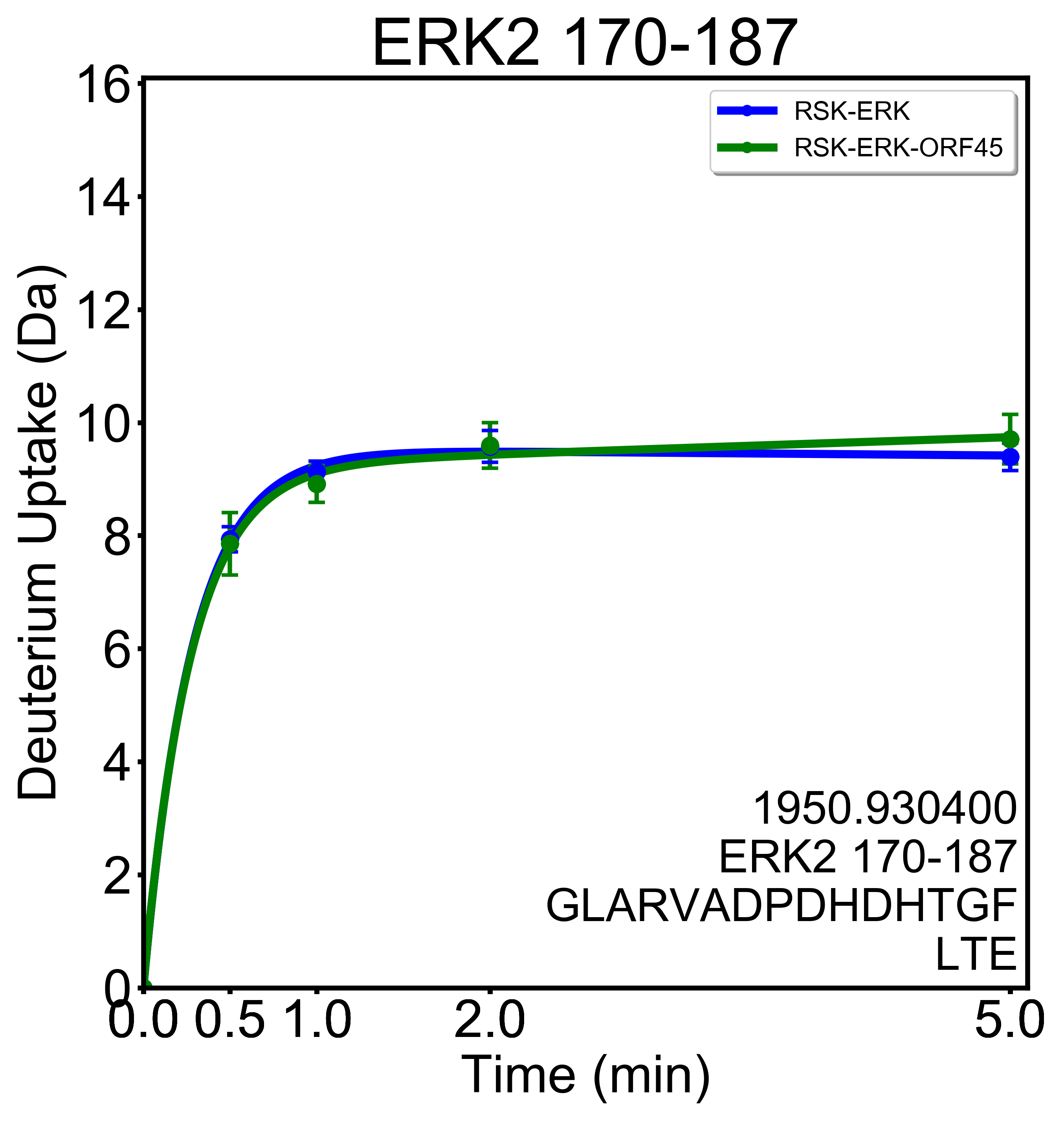 **ERK2**  β3-αC  Activation segment  Gly loop  Hinge  αF-αG  MAPK insert |
| --- |
| **Figure S3. Solvent protected and exposed regions of RSK2:ERK2 complex and RSK2:ERK2:ORF45 complex kinase domains.** (A) Uptake plots of solvent protected peptides for the ERK2, CTK, and NTK. (B) Uptake plots for higher uptake NTK peptides. (C) Uptake plots for higher uptake CTK peptides. (D) Uptake plots for higher uptake ERK2 peptides. |

| 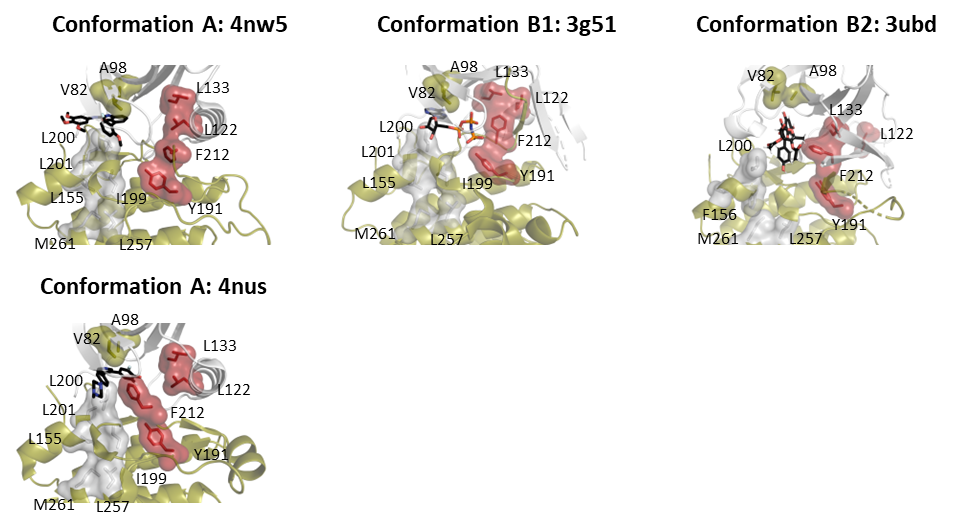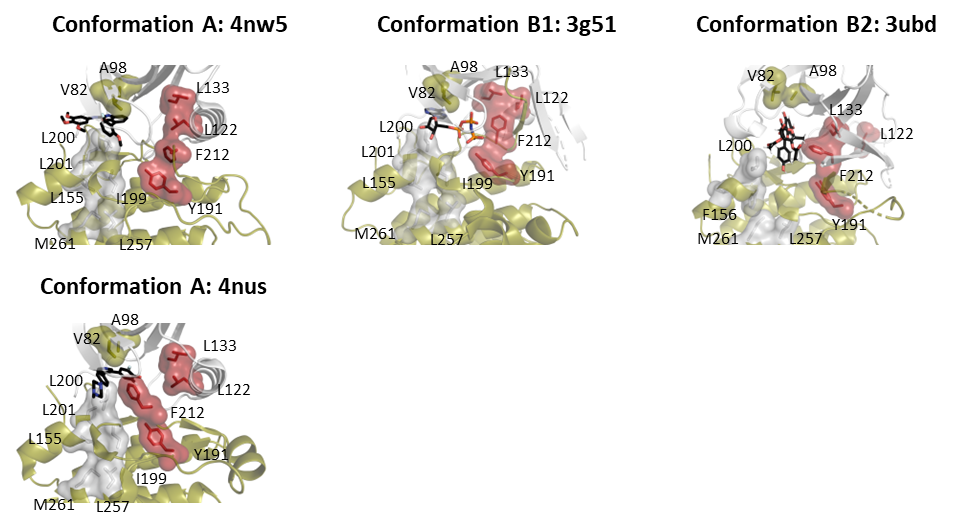 |
| --- |
| **Figure S4. Hydrophobic spines of the NTK in different N-lobe conformations.** Hydrophobic spines are displayed as gray and red surfaces for the C and R spines, respectively. |

| 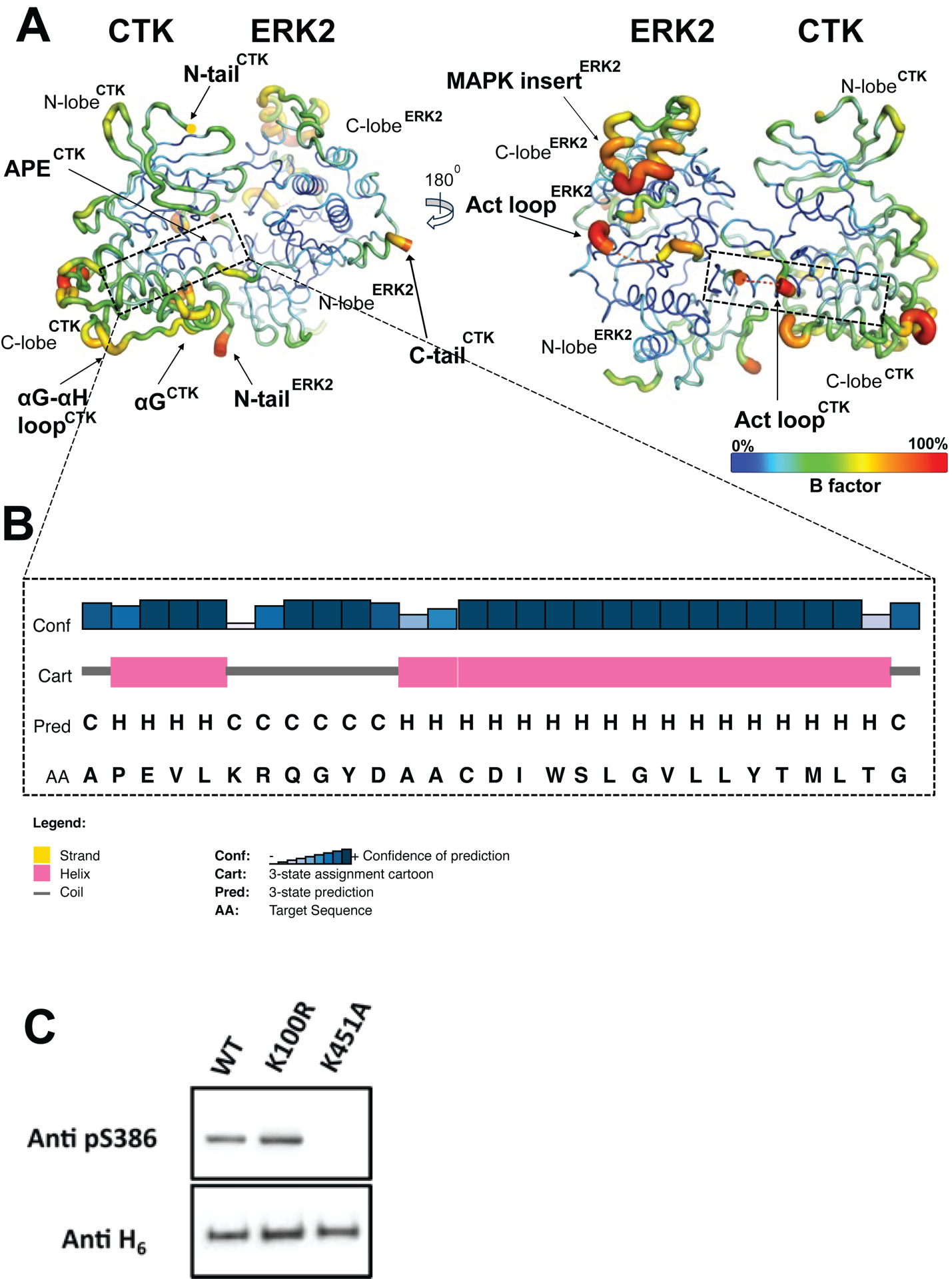 |
| --- |
| **Figure S5. CTK C-lobe flexibility and basal activity.** (A) CTK:ERK crystal structure (pdb: 4nif) colored by b-factors. (B) Secondary structure prediction of CTK from the APE motif throughout the αF helix. (C) Representative Western blot of FL RSK2 lysate (n=3) for WT, K100R (NTK kinase domain dead mutant), or K451A (CTK kinase domain dead mutant). The experiment was carried out in Ecoli overexpression system without the presence of ERK2 and PDK1. |

| 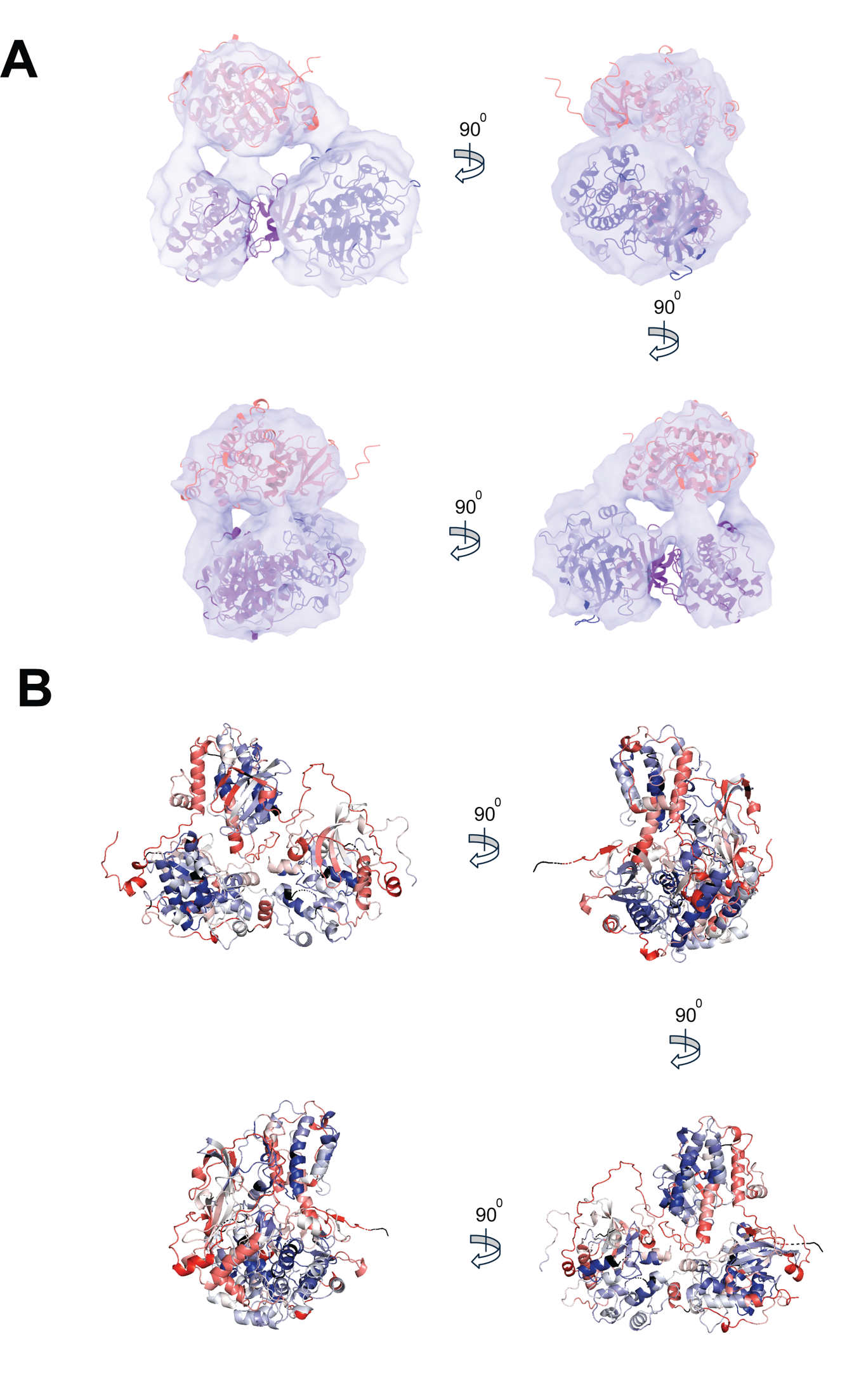 |
| --- |
| **Figure S6**: Different views of cryoEM map (A) and Alpha Fold models (B). (A) Each kinases domain was fitted into EM density lobe individually. Front and side views of cryoEM map were shown. (B) Front and side view of AlphaFold model with HDXMS data were shown. |

| 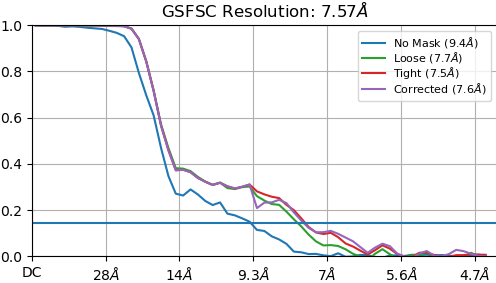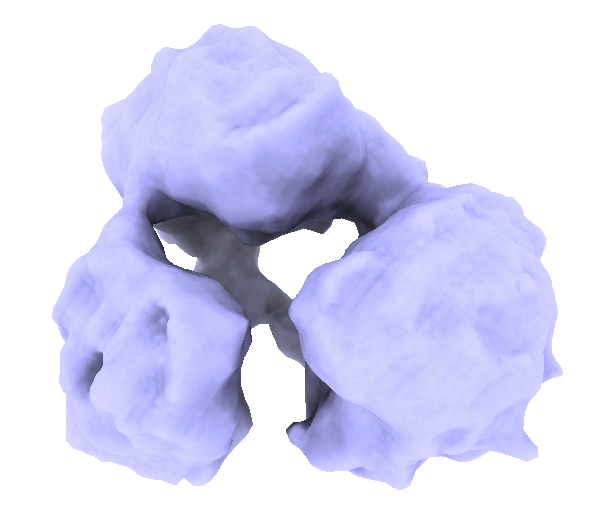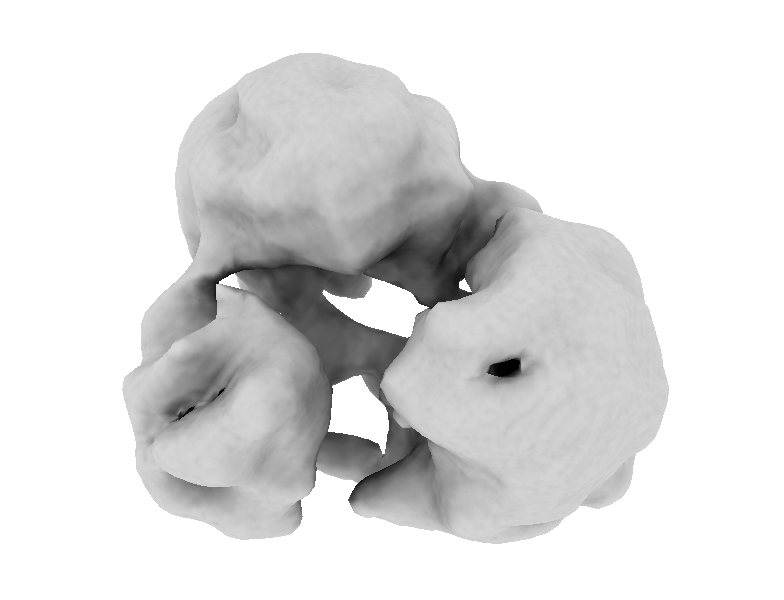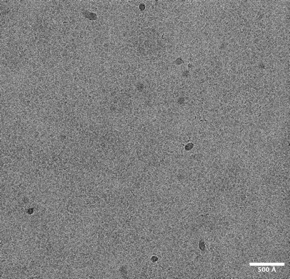  40,611 particles  Homo refinement  150 Å 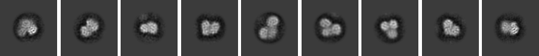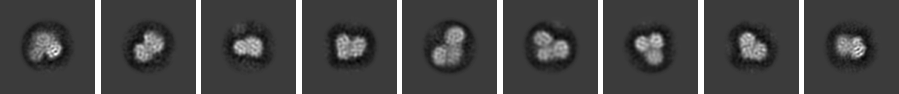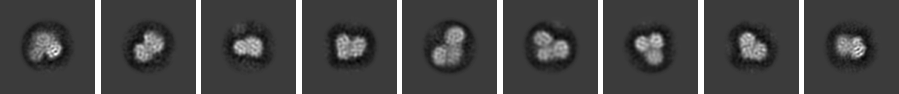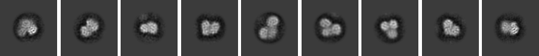 Ab-initio reconstruction  40,611 particles from multiple rounds of 2D classifications  Extract particles (256 -> 128 px)  (316,071 particles)  Particle picking  CTF cut 4.5 Å  cryoSPARC CTF Estimation  1025 micrographs  cryoSPARC Motion Correction |
| --- |
| **Figure S7**. CryoEM analysis workflow. Representative motion-corrected micrograph from data set. White scale bar is 500 Å. Representative 2D classes and reconstructed density and refined map are shown. |

| 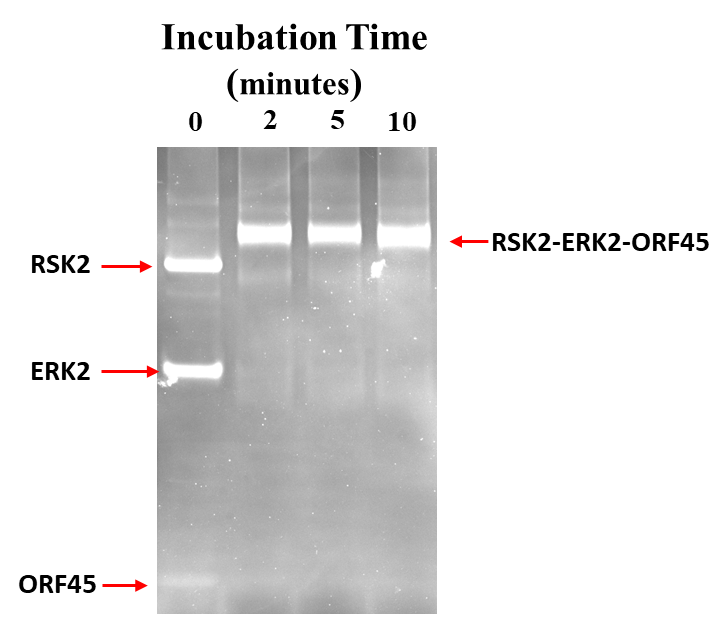  **RSK2:ERK2:ORF45**  **C**  **B**  **A** 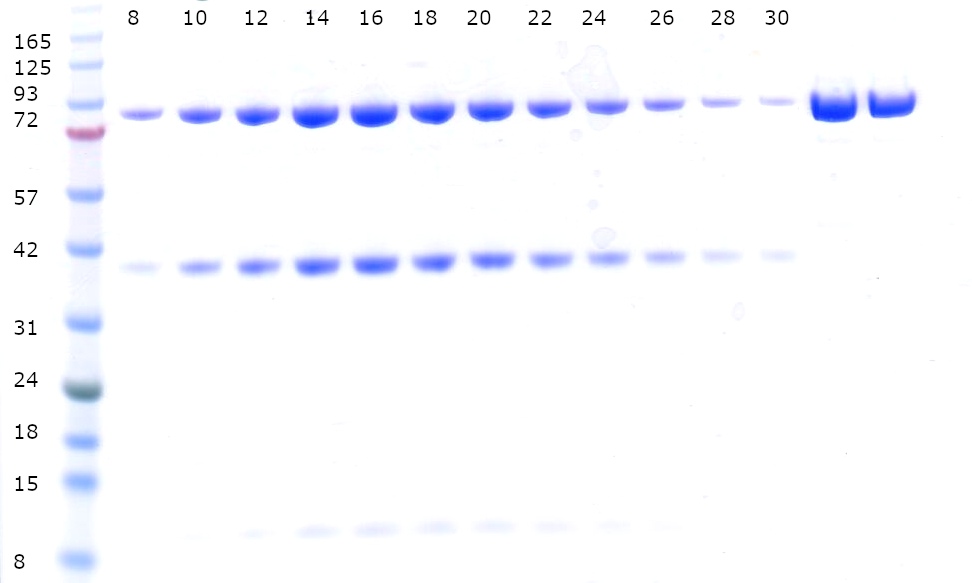 **ORF45**  **ERK2**  **RSK2** 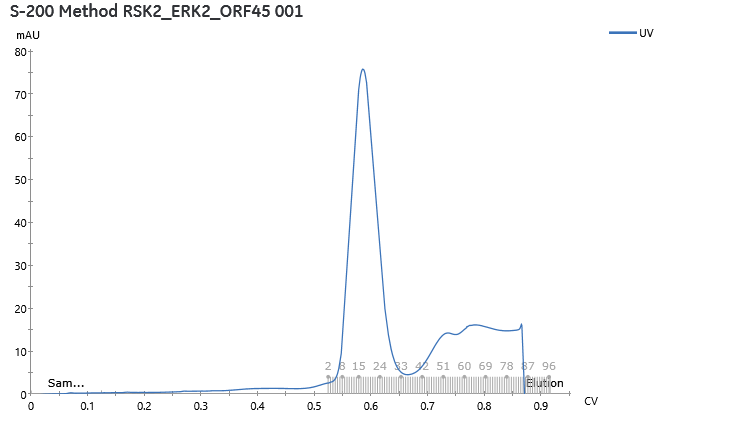 **RSK2:ERK2:ORF45**  **ERK2** |
| --- |
| **Figure S8**: Crosslinked RSK2:ERK2:ORF45 complex formation. (A) Representative size exclusion chromatogram of the RSK2:ERK2:ORF45 complex. (B) SDS-PAGE analysis of fractions corresponding to the complex peak. (C) SDS-PAGE of the crosslinking reaction of the RSK2:ERK2:ORF45 complex, stained with Sypro Ruby. Arrows indicate bands representing RSK2, ERK2, ORF45, and the crosslinked RSK2:ERK2:ORF45 complex. |
